# Effect of Parental Counseling on Infants’ Healthy Sleep Habits in Brazil: A Randomized Clinical Trial

**DOI:** 10.1001/jamanetworkopen.2019.18062

**Published:** 2019-12-20

**Authors:** Iná S. Santos, Bianca Del-Ponte, Luciana Tovo-Rodrigues, Camila S. Halal, Alicia Matijasevich, Suélen Cruz, Luciana Anselmi, Mariângela Freitas Silveira, Pedro R. Curi Hallal, Diego G. Bassani

**Affiliations:** 1 Postgraduate Program in Epidemiology, Federal University of Pelotas, Pelotas, Brazil; 2 Nossa Senhora da Conceição Children’s Hospital, Brazilian Ministry of Health, Porto Alegre, Brazil; 3 Departmento de Medicina Preventiva, Universidade de São Paulo, São Paulo, Brazil; 4 Department of Paediatrics, The Hospital for Sick Children, Research Institute, University of Toronto, Toronto, Ontario, Canada

## Abstract

**Importance:**

Poor sleep during early childhood is associated with adverse outcomes, including obesity, cognitive impairment, and mental and behavioral disorders.

**Objective:**

To assess the efficacy of an educational intervention in the promotion of nighttime sleep duration.

**Design, Setting, and Participants:**

This single-blind, intent-to-treat randomized clinical trial included participants in Pelotas, Brazil, aged 3 months who were followed up until age 24 months. Eligibility criteria included healthy infants aged approximately 3 months who slept less than 15 hours per 24 hours. Infants were randomized to the intervention group or control group.

**Interventions:**

Information on sleep characteristics, improvements in the environment, establishment of a nighttime sleep routine, and waiting before attending nocturnal awakenings was delivered to mothers in the intervention group by trained home-visitors at baseline. The intervention group received a telephone call on the first and second day after the intervention and a home visit on the third day after the intervention. The intervention’s content was reinforced at health care visits for ages 6 months and 12 months. Mothers allocated to the control group were counseled on the benefits of breastfeeding for the mother’s and child’s health and given written material with content on breastfeeding.

**Main Outcomes and Measures:**

Nighttime sleep duration was measured by interview and actigraphy at baseline and ages 6, 12, and 24 months and diaries at baseline and age 6 months. At ages 3 and 6 months, nighttime sleep self-regulation was calculated by subtracting nighttime sleep duration recorded by actigraphy from nighttime sleep duration recorded in the diaries and at ages 12 and 24 months by subtracting nighttime sleep duration recorded by actigraphy from nighttime sleep duration obtained by interview.

**Results:**

Among 1812 mother-infant dyads invited to participate, 798 met the inclusion criteria and 586 agreed to participate. The intervention group included 298 infants (154 [52.9%] boys), and the control group included 288 infants (164 [58.2%] boys). At age 6 months, mean (SD) nighttime sleep duration recorded in diaries was 9.80 (1.85) hours in the intervention group and 9.49 (2.07) hours in the control group, a difference of 19 minutes longer for the intervention group. At age 12 months, mean (SD) nighttime sleep duration based on the Brief Infant Sleep Questionnaire was 8.43 (1.35) hours in the intervention group and 8.52 (1.35) hours in the control group, a difference of 5 minutes shorter for the intervention group. At age 24 months, compared with information from the interview, actigraphy records showed that children in the intervention group stayed awake at night without signalizing for a mean (SD) of 0.52 (2.52) hours, whereas children in the control group stayed awake at night without signalizing for a mean (SD) of 0.23 (2.43) hours. There were no statistically significant difference between groups in any of the sleep parameters investigated.

**Conclusions and Relevance:**

This randomized clinical trial found that the educational intervention did not achieve longer nighttime sleep duration among infants in the intervention group.

**Trial Registration:**

ClinicalTrials.gov identifier: NCT02788630

## Key Points

**Question:**

Can an educational intervention improve infants’ nighttime sleep duration and sleep habits?

**Results:**

In this randomized clinical trial with 586 children assessed at ages 3 (baseline), 6, 12, and 24 months, mean nighttime sleep duration was 19 minutes longer in intervention group than the control group at age 6 months but 5 minutes shorter in intervention group than the control group at age 12 months. There were no statistically significant differences in the sleep parameters between the intervention group and control group at any age.

**Meaning:**

This randomized clinical trial found that the educational intervention did not achieve longer nighttime sleep duration among infants in the intervention group.

## Teaser

This randomized clinical trial examines the effect of parental counseling on bedtime routines and behaviors on infants’ sleep habits from ages 3 months to 2 years.

## Introduction

In recent decades, there has been a progressive decrease in the duration of sleep in Western populations, a pattern that starts in early infancy.^1^ Insufficient duration of sleep has been associated with various adverse health outcomes in childhood and adolescence, including impairments in cognitive development, learning and impulse control issues, behavioral issues, and metabolic and hormonal disruptions, which are associated with deficits in linear growth and obesity.^2,3,4,5,6,7,8^

Neurological maturation is a determinant factor for sleep patterns, especially in the process occurring in the first year of life.^9^ However, cultural differences are also key factors in the characterization of sleep in childhood.^10^ Behavioral interventions have proven effective in decreasing latency time,^11,12^ wake time,^13^ number of awakenings,^14,15,16,17,18^ and behavioral problems at bedtime,^19,20,21,22^ as well as increasing the duration of nighttime sleep,^23^ uninterrupted sleep,^24^ and sleep self-regulation. However, to our knowledge, none of these studies has been conducted in low- or middle-income countries, such as Brazil, where previous studies^10,25^ have reported different sleep habits in childhood compared with those seen in children in wealthy countries. Compared with children in high-income countries, children in Brazil have later bed and wake times by approximately 2 hours and have high levels of bedsharing with the mother.^10,25^

Thus, the main objective of this study was to test the efficacy of an intervention aimed to improve nighttime sleep among children aged 3 to 24 months. By improving sleep, the secondary objectives of the study were to promote child neurodevelopment and linear growth.

## Methods

The study protocol was approved by the institutional review board of the School of Medicine at the Federal University of Pelotas, affiliated with the Brazilian National Research Council. Before enrollment in the study, all mothers provided written informed consent. This study is reported following the Consolidated Standards of Reporting Trials (CONSORT) reporting guideline.

### Study Design and Eligibility Criteria

This was a parallel single-blind randomized clinical trial targeted to mothers of infants aged 3 months from the 2015 Pelotas Birth Cohort, a population-based study conducted in Pelotas, a city in southern Brazil. Detailed methods can be found in the published trial protocol^26^ and in the trial protocol in Supplement 1. Eligibility criteria included children born healthy (ie, without need of admission to a neonatal intensive care unit and without congenital malformations), from singleton pregnancies, gestational age at least 37 weeks, and who had slept a mean of less than 15 hours per 24 hours (daytime and nighttime sleep) during the previous week as reported by the mothers. Since the potential effect of scheduled maternal physical activity during pregnancy on sleep duration or on the child’s response to the intervention was not known, infants of mothers who participated in the PAMELA study^27^ were not eligible. Furthermore, because the intervention recommended a series of improvements in the home environment to ensure restorative sleep, the intervention was limited to families whose homes had at least 1 bedroom. Infants with continuous use of medications capable of altering sleep or causing sleepiness, such as anticonvulsants, were not eligible.

The trial protocol was approved by the funding agencies in May 2015, but owing to bureaucratic issues, the resources were available starting in May 2016. To guarantee the feasibility of the study, we started data collection in October 2015 without funding. In June 2016, when the resources needed for the completion of the trial were available, the trial registration was provided.

### Sample Size

Parameters used to calculate the sample size were 5% 2-tailed α with 80% power, a 1-to-1 ratio between the intervention group and control group, mean (SD) self-regulated sleep duration in the control group of 9.70 (1.98) hours at age 6 months and 10.25 (1.90) hours at age 12 months,^28^ and self-regulated sleep duration in the intervention group at least 30 minutes longer than in the control group at ages 6 and 12 months. Another 10% was added to the sample to compensate for potential losses to follow-up and adjusted analyses, so the final number was 276 children per study group.

### Randomization

Randomization was performed in blocks of 6. The results were stored in individual opaque envelopes that were numbered and sealed. For each child, the envelope was opened by the visitor only in the mother’s presence on the fifth day after she signed the informed consent form to participate in the study. The person who generated the random allocation sequence had no contact with the study participants.

### Intervention

Ten trained visitors delivered the intervention. The visitor training lasted 48 hours and consisted of a theoretical component on normal sleep in childhood and the intervention content, followed by role-playing exercises. The practical component, which occupied 40% of the training time, was conducted with mothers and infants attending outpatient clinics. Emphasis was given to the visitors’ communication skills (eg, asking questions, listening attentively to the mother’s answers, counseling using friendly language, praising the mother for things she was already doing to facilitate the child’s sleep, and asking checking questions to ascertain that the mother understood the advice). The training effectiveness was assessed at the end by applying a written test. Selection of the visitors was based on the test scores and assessment of the performance during the practical sessions.

The intervention was performed at the child’s home on the fifth day after the mothers signed the informed consent form and included information on sleep characteristics in the first year of life, improvements in the environment to promote falling asleep (eg, low noise levels and dim light), establishment of a nighttime sleep routine (eg, feeding, bathing, and putting to bed), putting the child to bed while still sleepy rather than when already asleep, and waiting 1 to 2 minutes before attending to the child during nocturnal awakenings.^29^

For mothers allocated to the intervention group, the visitor applied a checklist to assess the child’s sleep characteristics and the maternal strategies to deal with the child’s sleep. The visitor then made the recommendations addressing the topics that were relevant for the mother. A booklet containing the recommendations was given to the mothers in the intervention group at the end of the visit (eAppendix in Supplement 2). To promote maternal adherence, the visitor telephoned the mothers on the first and second days after the intervention and made a new visit on the third day after the intervention. The content of the intervention was reinforced during follow-up visits at ages 6 and 12 months. To prevent information bias in adherence to the recommendations, the reinforcement messages were only given after applying the questionnaire to assess the outcomes at ages 6 and 12 months.

### Control

For mothers allocated to the control group, the visitor counseled on the benefits of breastfeeding for the mother’s and child’s health and gave them written material with content on breastfeeding. Mothers from the control groups were visited at baseline (3 months after childbirth). Follow-up visits were conducted when children were ages 6, 12, and 24 months.

### Main Outcomes

Child sleep was assessed at baseline (age 3 months) and at ages 6, 12, and 24 months (Figure). Main outcomes were measured by the application of the Brief Infant Sleep Questionnaire (BISQ),^30,31^ diaries, and actigraphy. The BISQ was applied to the mothers in both groups during the baseline interview and at the visits at ages 6, 12, and 24 months. Mothers were instructed to report on the child’s sleep during the week prior to the interview.

**Figure.**
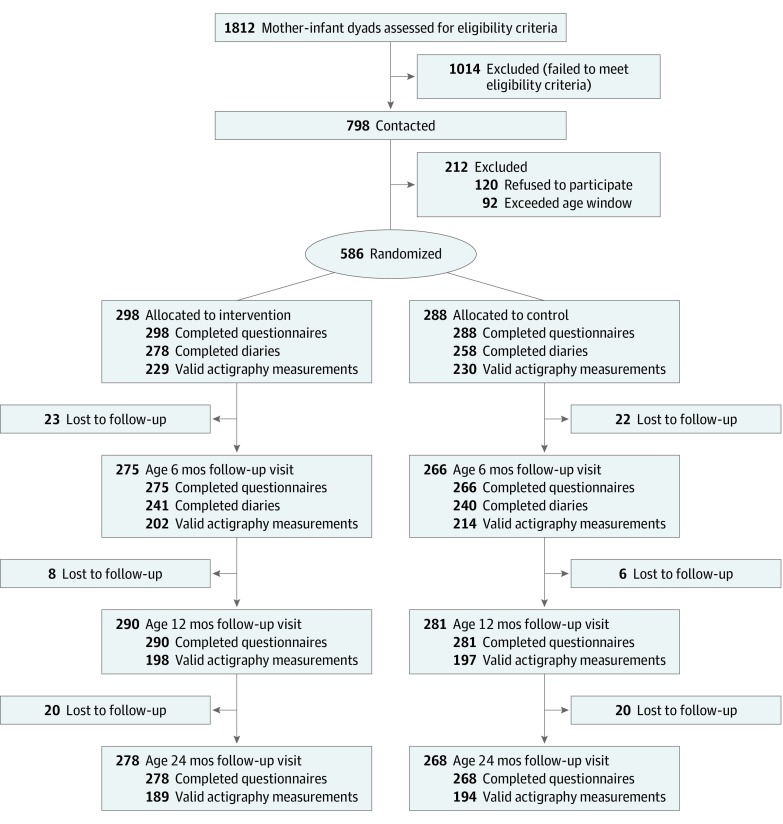
Study Flow Chart

A 24-hour activity diary was supplied to the mothers right after they signed the informed consent form (first day of the study) and at the visit at age 6 months (Figure). On both occasions, the mothers in the 2 groups completed the diaries for 5 days, recording the times in which the child was sleeping, awake, and being fed. During that time, the children wore an actigraphy monitor *(*wGT3X-BT monitor; ActiGraph) on the left ankle.^32^ Accelerometers were used at the age 3, 6, 12, and 24 months visits. The device was used for 5 days at ages 3 and 6 months and for 3 days at ages 12 and 24 months. At ages 3 and 6 months, records for at least 3 days were considered valid. At ages 12 and 24 months, records for at least 1 day were considered valid. Data were extracted using the Sadeh algorithm with 60-second epochs, having set 15 minutes as the minimum sleep period length and minimum wake time and 3 minutes for the bedtime definition.^33^ Children who fell asleep before 19:00 and woke up after this hour had this sleep period computed as daytime sleep. Likewise, children who woke up after 07:00 had this sleep computed as nighttime sleep.

The main outcomes were nighttime sleep duration and nighttime sleep self-regulation. Daytime sleep duration (between 07:00 and 19:00), nighttime sleep duration (between 19:00 and 07:00), 24-hour sleep duration, and number of nocturnal awakenings (defined by actigraphy as lasting ≥5 minutes) were obtained by 3 methods, interview, diaries, and actigraphy, at baseline and age 6 months. Nocturnal wake time, defined by BISQ as time awake between 22:00 hours and 06:00 and by actigraphy as time awake after first nighttime sleep onset, was obtained at baseline and at ages 6, 12, and 24 months. At ages 3 and 6 months, nighttime sleep self-regulation was calculated by subtracting nighttime sleep duration recorded by actigraphy from nighttime sleep duration recorded in the mothers’ diaries; and at ages 12 and 24 months by subtracting nighttime sleep duration recorded by actigraphy from nighttime sleep duration obtained by BISQ.

### Secondary Outcomes

Secondary outcomes included child anthropometry and neurodevelopment at ages 12 and 24 months. Children’s weight, length, weight-for-age, length-for-age, and body mass index–for-age *Z* scores at ages 12 and 24 months were obtained. Neurodevelopment was assessed at age 12 months with the Oxford Neurodevelopment Assessment tool and at age 24 months with the INTERGROWTH-21st Neurodevelopment Assessment tool.^34^

### Covariables

Perinatal covariables included family socioeconomic status, defined according to the Brazilian National Economic Index^35^ and divided into quintiles, with the first quintile as the lowest-resource group and the fifth quintile as the highest-resource group; maternal and paternal age and schooling (in complete years); maternal marital status (ie with or without partner); maternal number of previous pregnancies; maternal pregestational weight; maternal number of prenatal care visits; type of delivery; infant’s sex, infant birth weight, and infant 1- and 5-minute Apgar score. At age 3 months, information was collected on the infant’s age in days on the day of the visit, weight, length, breastfeeding pattern (ie, exclusive, predominant, partial, or weaned),^36^ principal caregiver (ie, mother or other person), bedsharing with mother (defined as habitual sharing of mother’s bed, either all night or part of the night and categorized as yes or no), number of siblings living in the same house, number of rooms used as bedrooms, and number of persons per bedroom. The mothers answered the Edinburgh Post-Natal Depression Scale^37^ in the version validated in Brazil.^38^

### Blinding

As this was an educational intervention, blinding was not possible for either the visitors or the mothers. However, because the interviewers who assessed the outcome did not know which group the child belonged to and because one of the outcome measures was objective, the study was single-blind.

### Statistical Analysis

The intervention and control groups were initially compared for baseline characteristics. At ages 6, 12, and 24 months, the intervention group and control group were compared in terms of differences in means or medians of sleep parameters with corresponding 95% CIs, after excluding outliers (eAppendix in Supplement 2). As the 2 groups were well balanced at baseline, only unadjusted analyses were performed. Main outcomes were analyzed cross-sectionally at each follow-up and longitudinally by generalized estimating equation models. All the analyses were intention-to-treat. The software package Stata version 13.1 (StataCorp) was used in the analyses. *P* values were 2-tailed, and statistical significance was set at *P* < .05.

## Results

Among 1812 children born in Pelotas, Brazil, from June 28 to December 31, 2015, and approached to participate in the study, 798 met all the eligibility criteria (Figure). Of these, 120 mothers refused to participate, and 92 infants exceeded the age window of 2.5 to 3.5 months stipulated for entering the trial. The mothers of the remaining 586 children were randomized to either the intervention group (298 infants; 154 [52.9%] boys) or the control group (288 infants; 164 [58.2%] boys). More than 93% of the children in both groups were assessed at all follow-ups.

At the time of childbirth, the parental mean (SD) age from the intervention group was 27.1 (6.6) years among mothers and 29.5 (8.2) among fathers, and the mean (SD) age in the control group was 27.4 (6.5) years among mothers and 29.9 (8.0) among fathers (Table 1). At baseline, most of the mothers were the main caregiver of the child (intervention group: 286 mother-infant dyads [96.3%]; control group: 278 mother-infant dyads [96.9%]), most of the infants were exclusively breastfed (intervention group: 141 mother-infant dyads [47.3%]; control group: 127 mother-infant dyads [44.3%]), and approximately half usually bedshared with the mother the whole night or part of the night (intervention group: 138 mother-infant dyads [47.6]; control group: 122 mother-infant dyads [44.2%]).

**Table 1.** Baseline Characteristics of Infants

Characteristic	Infants, No. (%)	
	Intervention Group (n = 298)	Control Group (n = 288)
IEN, quintile^a^		
First	55 (19.0)	52 (18.3)
Second	69 (23.9)	62 (21.8)
Third	52 (20.8)	52 (18.3)
Fourth	65 (22.9)	65 (22.9)
Fifth	53 (18.7)	53 (18.7)
Education, mean (SD), y		
Maternal	10.1 (3.7)	10.1 (3.8)
Paternal	8.9 (3.9)	9.4 (4.1)
Age, mean (SD), y		
Maternal	27.1 (6.6)	27.4 (6.5)
Paternal	29.5 (8.2)	29.9 (8.0)
Pregnancies, mean (SD), No.	0.7 (1.0)	0.8 (1.1)
Pregestational maternal weight, mean (SD), kg	68.1 (16.0)	71.6 (57.2)
Antenatal visits, mean (SD), No.	9.1 (2.7)	8.9 (2.8)
Type of delivery		
Vaginal	120 (40.3)	102 (35.5)
Cesarean	178 (59.7)	185 (64.5)
Infant’s sex		
Boy	154 (52.9)	164 (58.2)
Girl	137 (47.1)	118 (41.8)
Birth weight, mean (SD), g	3275.87 (443.06)	3274.95 (454.11)
Apgar score, mean (SD), min		
1	8.8 (5.4)	8.6 (1.4)
5	9.9 (5.2)	9.5 (0.7)
Age at 3 mo, mean (SD), d	91.4 (2.6)	91.3 (2.6)
Weight at 3 mo, mean (SD), g	6200.8 (767.6)	6283.2 (823.9)
Length at 3 mo, mean (SD), cm	60.6 (2.3)	60.5 (2.5)
Breastfeeding pattern		
Exclusive	141 (47.3)	127 (44.3)
Predominant	19 (6.4)	21 (7.3)
Partial	67 (22.5)	77 (26.8)
Weaned	71 (23.8)	62 (21.6)
Mother as principal caregiver	286 (96.3)	278 (96.9)
Mother lives with husband or companion	253 (86.9)	230 (81.6)
Bedsharing with mother	138 (47.6)	122 (44.2)
Siblings living in same house, No.		
0	153 (52.6)	142 (50.3)
1	99 (34.0)	88 (31.2)
≥2	39 (13.4)	52 (18.4)
Bedrooms in home, mean (SD), No.	1.8 (0.8)	1.8 (0.7)
Persons per bedroom, mean (SD), No.	2.6 (0.8)	2.7 (1.1)
Maternal EPDS score, mean (SD)	5.8 (4.9)	5.2 (4.6)

Abbreviations: EPDS, Edinburgh Post-Natal Depression Scale; IEN, Brazilian National Economic Index.

^a^ First indicates the lowest-resource quintile; fifth, highest-resource quintile.

At baseline, mean (SD) total sleep duration was 12.18 (1.53) hours in the intervention group and 12.27 (1.45) in the control group, and mean (SD) nighttime sleep duration was 8.05 (1.28) hours in the intervention group and 7.97 (1.22) hours in the control group, as reported by the mothers (Table 2). The median (interquartile range [IQR]) number of nocturnal awakenings was 1.0 (0-2.0) in the intervention group and 1.0 (1.0-2.0) in the control group. The median (IQR) nocturnal wake time duration was 1.50 (1.00-2.00) hours in the intervention group and 1.42 (0.50-2.00) hours in the control group. As recorded in the diaries, mean (SD) total sleep duration was 12.70 (2.63) hours in the intervention group and 12.78 (2.17) hours in the control group, and mean (SD) nighttime sleep duration was 9.47 (2.37) hours in the intervention group and 9.53 (2.07) hours in the control group. The median (IQR) number of nocturnal awakenings was 2.0 (1.3-2.7) in the intervention group and 2.0 (1.3-3.0) in the control group (Table 2). In actigraphy records, mean (SD) total sleep duration was 11.22 (1.73) hours in the intervention group and 10.98 (1.53) hours in the control group, and mean (SD) nighttime sleep duration was 8.13 (1.53) hours in the intervention group and 8.03 (1.48) hours in the control group. The median (IQR) number of nocturnal awakenings was 3.0 (2.3-4.0) in the intervention group and 3.3 (2.5-4.0) in the control group. The median (IQR) nocturnal wake time duration was 1.20 (0.50-2.33) hours in the intervention group and 1.22 (0.60-2.27) in the control group (Table 2). A total of 36 mothers considered their infant’s sleep habits a problem at baseline, including 17 mothers (5.7%) in the intervention group and 19 mothers (6.6%) in the control group.

**Table 2.** Baseline Sleep Characteristics

Characteristic	Intervention Group	Control Group
**Reported by Interview **		
No.	298	288
Sleep duration, mean (SD), h		
Total	12.18 (1.53)	12.27 (1.45)
Daytime	4.13 (1.53)	4.32 (1.35)
Nighttime	8.05 (1.28)	7.97 (1.22)
Nocturnal awakenings, median (IQR), No.	1.0 (0-2.0)	1.0 (1.0-2.0)
Nocturnal wake time duration, median (IQR), h^a^	1.50 (1.00-2.00)	1.42 (0.50-2.00)
**Reported by Diary**^b^		
No.	278	258
Sleep duration, mean (SD), h		
Total	12.70 (02.63)	12.78 (2.17)
Daytime	3.22 (1.22)	3.23 (1.12)
Nighttime	9.47 (2.37)	9.53 (2.07)
Nocturnal awakenings, median (IQR), No.	2.0 (1.3-2.7)	2.0 (1.3-3.0)
**Reported by Actigraphy**^b^		
No.	229	230
Sleep duration, mean (SD), h		
Total	11.22 (1.73)	10.98 (1.53)
Daytime	3.12 (0.88)	3.00 (0.78)
Nighttime	8.13 (1.53)	8.03 (1.48)
Nocturnal awakenings, median (IQR), No.	3.0 (2.3-4.0)	3.3 (2.5-4.0)
Nocturnal wake time duration, median (IQR), h^a^	1.20 (0.50-2.33)	1.22 (0.60-2.27)

Abbreviation: IQR, interquartile range.

^a^ Nocturnal wake time defined as from 22:00 to 06:00.

^b^ Reported as a mean of 3 days.

### Main Outcomes

Fidelity of visitors in the delivery of the intervention (eTable 1 in Supplement 2) and maternal adherence with the recommendations (Table 3) were assessed at the age 6 months visit. Among the intervention group, 35 mothers (12.7%) reported putting the child to bed while drowsy but awake rather than already asleep, while 29 mothers (10.9%) in the control group reported this practice. Additionally, 64 mothers (24.1%) reported waiting 1 to 2 minutes before attending nocturnal awakenings, compared with 7 mothers (2.8%) in the control group.

**Table 3.** Comparison of Adherence in Intervention and Control Groups for Practices Recommended by the Intervention at Age 6 Months

Maternal Practice	No. (%)		Difference, % (95% CI)
	Intervention Group (n = 275)	Control Group (n = 266)	
Put the child drowsy but awake in the bed instead of sleeping	35 (12.7)	29 (10.9)	1.8 (−3.7 to 7.3)
Infant sleeps in supine position	135 (49.1)	112 (42.1)	7.0 (0.8 to 13.2)
Infant sleeps in crib next to mother’s bed	155 (56.4)	124 (46.6)	9.8 (1.4 to 18.2)
Bedtime routine	191 (69.5)	163 (61.3)	8.2 (0.2 to 16.2)
Mother waits 1-2 min before attending nocturnal awakenings	64 (24.1)	7 (2.8)	21.3 (14.2 to 28.4)
Decorations in infant’s crib	115 (48.7)	141 (62.7)	−14.0 (−22.4 to −0.06)
Bedsharing with mother	138 (47.6)	121 (44.2)	3.4 (−5.0 to 11.8)
Calm household environment at night	256 (93.1)	255 (95.9)	−2.8 (−6.5 to 0.9)

According to maternal reports, there was no difference between the intervention group and control group in terms of sleep parameters (Table 4). Mean (SD) nighttime sleep duration was 8.99 (1.73) hours in the intervention group and 8.98 (1.67) hours in the control group at age 6 months and 8.43 (1.35) hours in the intervention group and 8.52 (1.35) hours in the control group at age 12 months. At age 24 months, children from the intervention group had a mean (SD) nocturnal sleep duration of 7.61 (1.95) hours and those from the control group had a mean (SD) nocturnal sleep duration of 7.88 (2.02) hours.

**Table 4.** Sleep Outcomes at Ages 6, 12, and 24 Months

Outcome	Age								
	6 mo			12 mo			24 mo		
	Intervention Group	Control Group	*P* Value	Intervention Group	Control Group	*P* Value	Intervention Group	Control Group	*P* Value
**Reported by Maternal Interview **									
No.	275	266		290	281		278	268	
Sleep duration, mean (SD), h									
Total	12.10 (2.13)	12.20 (2.13)	.60^a^	11.65 (1.85)	11.55 (1.68)	.51^a^	10.81 (2.07)	11.07 (1.96)	.12^a^
Daytime	3.23 (1.61)	3.33 (1.78)	.45^a^	3.22 (1.58)	3.03 (1.43)	.17^a^	3.00 (1.58)	3.13 (1.45)	.28^a^
Nighttime	8.99 (1.73)	8.98 (1.67)	.99^a^	8.43 (1.35)	8.52 (1.35)	.47^a^	7.61 (1.95)	7.88 (2.02)	.34^a^
Nocturnal awakenings, median (IQR), No.	1.0 (1.0-3.0)	2.0 (1.0-3.0)	.09^b^	1.0 (0-2.0)	0 (0-2.0)	.81^b^	0 (0-1.0)	0 (0-2.0)	.53^b^
Nocturnal wake time duration, median (IQR), h^c^	0.42 (0.08-1.00)	0.50 (0.08-1.00)	.87^b^	0.50 (0-1.50)	0.50 (0-1.50)	.81^b^	0.50 (0-1.50)	0.50 (0-1.25)	.57^b^
**Reported by Diaries**^d^									
No.	241	240		NA	NA		NA	NA	
Sleep duration, mean (SD), h									
Total	12.24 (2.01)	12.04 (2.29)	.33^a^	NA	NA	NA	NA	NA	NA
Daytime	2.40 (1.05)	2.48 (1.10)	.40^a^	NA	NA	NA	NA	NA	NA
Nighttime	9.80 (1.85)	9.49 (2.07)	.10^a^	NA	NA	NA	NA	NA	NA
Nocturnal awakenings, median (IQR), No.	2.0 (1.0-2.7)	1.7 (1.0-2.7)	.90^b^	NA	NA	NA	NA	NA	NA
**Reported by Actigraphy**^**e**^									
No.	202	214		198	197		189	194	
Sleep duration, mean (SD), h									
Total	11.52 (1.46)	11.46 (1.93)	.72^a^	10.23 (1.79)	10.19 (1.75)	.81^a^	10.22 (1.68)	10.18 (1.76)	.83^a^
Daytime	2.73 (1,25)	2.78 (1.31)	.67^a^	2.27 (1.16)	2.24 (1.11)	.82^a^	1.92 (1.35)	1.73 (1.13)	.14^a^
Nighttime	8.76 (1.32)	8.59 (1.33)	.22^a^	7.99 (1.52)	8.02 (1.93)	.85^a^	8.17 (1.42)	8.32 (1.39)	.33^a^
Nocturnal awakenings, median (IQR), No.	2.3 (1.7-3.0)	2.0 (1.7-3.0)	.49^b^	2.0 (2.0-3.0)	3.0 (2.0-4.0)	.20^b^	2.0 (1.0-3.0)	2.0 (1.0-3.0)	.37^b^
Nocturnal wake time duration, median (IQR), h^c^^,^^f^	0.57 (0-1.18)	0.71 (0.07-1.51)	.18^b^	0.91 (0-1.98)	1.10 (0-2.28)	.54^b^	0.64 (0-1.58)	0.23 (0-1.55)	.16^b^

Abbreviations: IQR, interquartile range; NA, not available.

^a^ Calculated with *t* test.

^b^ Calculated with Mann-Whitney test.

^c^ Nocturnal wake time defined as from 22:00 to 06:00.

^d^ Reported as a mean of 3 days.

^e^ Reported as a mean of 3 days at age 6 months and as 1 day of data at ages 12 and 24 months.

^f^ Nocturnal wake time measured after first sleep onset.

As recorded in the diaries at 6 months, mean (SD) nighttime sleep duration was approximately 19 minutes longer in the intervention group than in the control group (Table 4), with mean (SD) nighttime sleep duration of 9.80 (1.85) hours in the intervention group and 9.49 (2.07) hours in the control group.

As in baseline, nighttime sleep duration measured by actigraphy at ages 6, 12, and 24 months was shorter than that reported by the mothers, while the median number of awakening episodes was greater (Table 4). At age 6 months, the mean (SD) nighttime sleep duration as recorded by actigraphy was 8.76 (1.32) hours with a median (IQR) of 2.3 (1.7-3.0) nocturnal awakenings in the intervention group and 8.59 (1.33) hours with a median (IQR) of 2.0 (1.7-3.0) nocturnal awakenings in the control group. At age 12 months, mean (SD) nighttime sleep duration was 7.99 (1.52) hours with a median (IQR) of 2.0 (2.0-3.0) nocturnal awakenings in the intervention group and 8.02 (1.93) hours with a median (IQR) of 3.0 (2.0-4.0) nocturnal awakenings in the control group. At age 24 months, mean (SD) nighttime sleep duration was 8.17 (1.42) hours with a median (IQR) of 2.0 (1.0-3.0) nocturnal awakenings in the intervention group and 8.32 (1.39) hours with a median (IQR) of 2.0 (1.0-3.0) nocturnal awakenings in the control group, meaning that except for the control group at age 24 months, median nocturnal wake time duration as measured by actigraphy was greater than that reported by the mothers at the interview.

There were no statistically significant differences between groups at any age, regardless of the method used to measure the sleep parameters. Similarly, there was no difference between groups at any age in terms of nighttime sleep self-regulation. For instance, at age 6 months, comparing the records from diaries with those from actigraphy, the time the infants stayed awake without being noticed by their mothers was very similar at the 2 groups (intervention group, 0.98 [95% CI, 0.98-1.31] hours; control group, 0.95 [95% CI, 0.59-1.32] hours). At age 24 months, compared with information from the interview, actigraphy records showed that children in the intervention group stayed awake at night without signalizing for a mean (SD) of 0.52 (2.52) hours, whereas children in the control group stayed awake at night without signalizing for a mean (SD) of 0.23 (2.43) hours. In longitudinal analyses, there was no statistically significant difference between the groups in nighttime sleep duration, 24-hour sleep duration, nocturnal wake time, or number of nocturnal awakenings as assessed by BISQ or actigraphy.

Exploratory analyses were conducted in a subgroup of children who slept less than recommended at age 3 months (ie, <11 hours total per day).^39^ This criterion was met by 49 children in the intervention group and 41 in the control group. According to actigraphy at age 12 months, the median number of nocturnal awakenings was significantly lower in the intervention group (median [IQR], 2.0 [1.0-3.0] awakenings per night) than in the control group (median [IQR], 3.0 [2.0-4.5] awakenings per night; *P* = .001).

### Secondary Outcomes

At ages 12 and 24 months, there were no statistically significant differences between groups on any of the target growth parameters (eTable 2 and eTable 3 in Supplement 2). As for neurodevelopment at age 12 months, there was a statistically significant difference in mean (SD) scores in cognitive domain among children in the intervention group compared with children in the control group (67.9 [11.2] vs 65.8 [10.5]; difference, 2.1 [95% CI, 0.3-4.0]; *P* = .02) (eTable 2 in Supplement 2).

## Discussion

This randomized clinical trial found that despite the fact that nighttime sleep duration at age 6 months as recorded by the mothers in the diaries was approximately 19 minutes longer in the intervention group and nighttime sleep self-regulation at age 24 months was 31 minutes in the intervention group vs 14 minutes in the control group, maternal counseling by trained visitors 3 months after childbirth did not significantly improve children’s sleep quality or duration at ages 6, 12 or 24 months. The fact that the children’s nighttime sleep duration at baseline was within the normal range for their age is among the possible reasons for the intervention’s lack of success.^39^ Most of the previously published interventions were targeted to children with severe sleeping problems^11,12,13,14,16,18,19,20,22,40,41^ and thus higher probability of benefiting from the intervention.^42^ The children in our sample were from the general population, and at baseline, only approximately 6% of the mothers from each group considered their infant’s sleep a problem. When we limited the analysis to the children who slept less than the lower limit of normal at age 3 months,^39^ the number of nocturnal awakenings at 12 months was lower in intervention group than in the control group.

Of nearly 24 interventions planned to improve child sleep that have been previously published, only 3 did not find a statistically significant difference between the intervention and control groups.^40,41,43^ In addition, only 2 interventions assessed sleep objectively through actigraphy,^43^ and only 1 found benefit from the intervention on the children’s sleep.^14^ Most of the successful interventions focused on consistent bedtime routine and the Ferber method (also known as *graduated extinction*). In our study, although healthy sleep practices were reported 3 months after the intervention more frequently by mothers from the intervention group than from the control group, only 24% of the mothers from the intervention group adhered to the recommendation of waiting 1 to 2 minutes before attending nocturnal wakings, and 13% of mothers adhered to the recommendation of putting the child drowsy but awake in the bed instead of asleep.

Worthy of note was the difference identified in our study between the 2 groups as to neurodevelopment at age 12 months. Sleep plays a prime role in children’s cognitive, social, and emotional development.^44^ However, in our study, children’s sleep at age 12 months had no effect on cognitive abilities; thus, this result cannot be interpreted as an effect of the intervention.

### Strengths and Limitations

Our study has some noteworthy strengths. Concerning the method for assessing the outcome, studies that rely only on the parents’ report of children’s sleep duration generally cannot distinguish between time in bed and real sleep time. Real sleep time is typically shorter than time in bed, and the parents’ report thus tends to overestimate sleep duration. Meanwhile, intervention studies using sleep duration measurements in the laboratory (outside the children’s habitual environment and generally measuring sleep on a single night) typically produce shorter sleep periods. Studies that have used both subjective and objective methods have found that parents usually underestimate the child nocturnal wake time duration. The actigraph continuously records and sums the number of limb movements for a given period. Then, through the use of a specially developed algorithm, the motility levels are translated into states of sleep or wakefulness.^45^ In addition to interviews, we used maternal records (ie, diaries) and actigraphy to measure and compare sleep habits before and after performing the intervention. These are reliable methods, recommended to assess the effect of interventions to improve children’s sleep. Another strength of the study was the efficiency of randomization.^46^ Our study measured a series of factors associated with sleep in childhood, and these factors were well balanced between the 2 groups. The low rate of children’s losses to follow-up was another strength in the study.

Our study also has some limitations. Traditionally, nighttime sleep self-regulation is measured either by videosomnography or by direct observation.^28^ In our study, to estimate duration of quiet wakefulness we used the difference between waking time by actigraphy and by diary records or maternal report. Our study is limited by the fact that parents would only record or report periods of signaling wakefulness, thus missing periods of quiet wakefulness. Additionally, considering that actigraphy generally overestimates the duration of wakefulness periods,^47^ the differences in nighttime sleep self-regulation we observed in both groups are likely overestimated. Moreover, a 1-day valid record of sleep using actigraphs at ages 12 and 24 months is another limitation of the study because sleep variability is important at these ages.^48^

Since this was an efficacy study, maternal adherence to the study protocol was essential to achieve the objectives. Low maternal adherence to the recommended practices would be a potential limitation to this study. The assessment of maternal adherence at infant age 6 months follow-up showed a higher proportion of mothers in the intervention group than in the control group reporting practicing the specific recommendations.

## Conclusions

In this randomized clinical trial, the pathway linking the success of a sleep intervention with improved child nighttime sleep included a sequence of events, starting with the visitors’ adequate knowledge on how to inquire about sleep in childhood and on counseling mothers on ways to improve their child’s nighttime sleep, followed by maternal adherence to the practices recommended by the visitors, and methodological characteristics of the study (eg, efficient randomization, objective assessment of sleep, low rate of losses to follow-up, and intention-to-treat analysis). Despite the fact that most of these steps had been successfully achieved in our study, the intervention did not improve nighttime sleep duration or nighttime sleep self-regulation among children in the intervention group.

## Notes

Supplement 1. **Trial Protocol**

Supplement 2. **eAppendix.** Supplemental Methods **eTable 1.** Maternal Recall at 6 Months After Childbirth of the Advice Received at Baseline **eTable 2.** Effect of Intervention on Growth and Development at Age 12 mos **eTable 3.** Effect of Intervention on Growth and Development at Age 24 mos

Supplement 3. **Data Sharing Statement**
