# Supplementary material for: Effect of Parental Counseling on Infants’ Healthy Sleep Habits in Brazil: A Randomized Clinical Trial
Source: JAMA Netw Open. 2019 Dec 20;2(12):e1918062. doi: 10.1001/jamanetworkopen.2019.18062 (PMC6991232; doi:10.1001/jamanetworkopen.2019.18062)
Supplement: Supplement 1. — Trial Protocol [file jamanetwopen-2-e1918062-s001.pdf]

INFANT SLEEP HYGIENE COUNSELING: A RANDOMIZED CONTROLLED TRIAL

Research Project

In response to RFP Nº 47/2014

MCTI/CNPq/MS/SCTIE/Decit/ Bill & Melinda Gates Foundation

Investigators:

Iná S. Santos[1]

Diego G. Bassani[2]

Alicia Matijasevich[3]

Camila S. Halal[4]

Luciana Anselmi[1]

Elaine Albernaz[5]

Michele Fernandes[6]

Mariângela F. Silveira[1]

Pedro C. Hallal[1]

Institutions

[1]Programa de Pós-graduação em Epidemiologia - Universidade Federal de Pelotas, Brasil

[2]The Hospital for Sick Children - Research Institute and Department of Paediatrics, University of Toronto, Canada

[3]Departamento de Medicina Preventiva – Universidade de São Paulo, Brasil

[4]Hospital da Criança Conceição – Ministério da Saúde, Porto Alegre, RS, Brasil

[5]Departamento de Saúde materno-Infantil, Universidade Federal de Pelotas, Brasil

[6]Oxford Maternal & Perinatal Health Institute International Research Fellow, Nuffield Department of Obstetrics & Gynaecology, John Radcliffe Hospital, University of Oxford & Clinical Fellow, Department of Paediatrics, Southampton General Hospital, United Kingdom

Pelotas, Brazil, May 2015

ABSTRACT

The development of adequate sleep patterns is paramount to the optimal neurodevelopment, as well as to promote proper learning, develop memory and preserve cerebral plasticity. Factors that hinder sleep patterns can negatively influence the child's growth, neurological and motor development. This study will evaluate the efficacy of an intervention to improve quality and duration of self-regulated nighttime sleep (the amount of time the child maintains a combination of uninterrupted sleep, quiet wakefulness, and re-initiation of sleep without parental intervention). We will conduct a randomized controlled trial within the 2015 Pelotas Birth Cohort. At the 3-month follow-up, children who sleep less than 13 hours per 24 hours (nighttime sleep and daytime naps) will be eligible for the study. The sample size was estimated at 276 children per arm. Nighttime self-regulated sleep duration will be evaluated at baseline (age 3 months) and at 6, 12 and 24 months using actigraphy and via a sleep diary completed by the mother. Following block randomization, mothers from the intervention group will be visited by a trained fieldworker who will deliver counseling on age-appropriate normal sleep behaviors, how to facilitate sleep onset and how to manage night awakenings.

I) INTRODUCTION

Human capital is the body of knowledge, skills, competencies and other characteristics that promote personal, social and economical well-being. There is growing evidence from life-course research, especially from birth cohort studies, supporting the role of early life factors on the human capital(1, 2). One of the widely prevalent, but relatively poorly understood, life factors affecting human capital during both childhood and later life is the quality and duration of sleep. About 1 in 4 children aged under 5 years of age suffer from sleep problems, commonly presented as nighttime awakening and difficulty falling asleep. Additionally, developmental disturbances such as enuresis, somnambulism and encopresis, linked with childhood anxiety and stress; and childhood neurological conditions such as sleep apnea are part of the spectrum of childhood sleep disturbances. Childhood sleep problems have been found to be associated with memory and learning impairments, irritability, difficulties in mood modulation, attention and behavioral problems (including aggressive behavior), hyperactivity and impulsivity.(3-7)

In addition to contributing to developmental problems, chronic sleep problems can promote metabolic and hormonal disruptions resulting in deficits in linear growth.(8) The human Growth Hormone (GH), responsible for child growth, especially linear growth, has its maximum expression approximately one hour after sleep onset.(9) When the secretion of GH is impaired due to less efficient sleep patterns (total sleep time/total time in bed) or due to reductions in sleep duration there can be an increase in the weight:height ratio from both increases in weight but also due to reductions in linear growth.(10) Interventions that included activities to promote quality and increase in duration of child sleep showed benefits in linear growth. (11, 12) Sleep quality, measured using actigraphy (percent of motionless sleep time) was associated with greater linear growth at 6 months.(10)

By targeting an essential component of infant development, i.e. sleep, this proposal responds to one of the main objectives of this call, to identify effective interventions to reduce growth deficit and to promote cognitive and neurological development. The present proposal is nested within the 2015 Pelotas Birth Cohort, currently underway. In addition, this study is innovative since there are no other reports in the literature about sleep interventions in middle-income countries such as Brazil.

II) JUSTIFICATION

Insufficient sleep is associated with a number of adverse health outcomes in childhood, including impaired cognition, diminished impulse control, behavioral problems, and obesity.(13-18) The United States National Sleep Foundation recommends 10.5 to 18 hours of sleep within a 24-hour period for infants, and 11-13 hours within 24 hours for preschoolers.(19) In 2011, the Institute of Medicine (IOM) recommended a number of simple but effective 'action points' to achieve these goals: (1) create environments that ensure restful sleep (no screen media in rooms where children sleep, and low noise and lighting levels during napping); (2) encourage sleep-promoting behaviors and practices, such as calming naptime routines and avoiding stimulating or stressing children just before naptime; and (3) encourage practices that promote child self-regulation of sleep, including putting infants to sleep drowsy but awake and helping older children to identify feelings of sleepiness.(20)

A number of population-based studies have established that sleep problems occur more frequently among infants and children from households with financial difficulties than those without financial difficulties (21, 22). Therefore, sleep-promoting interventions tailored toward socially disadvantaged children may help to (i) promote good quality sleep and (ii) prevent or minimize the negative developmental consequences of poor sleep in these children.

Furthermore, because the study will be carried out with children from a birth cohort that will continue to be followed, we will be able to evaluate outcomes later in life such as attained height, academic achievement, IQ and economic productivity in adult life.

III) LITERATURE REVIEW

1) SLEEP IN THE FIRST YEAR OF LIFE

Infants experience rapid changes in their capacity for self-regulating nocturnal sleep and to sustain sleep without demanding parental assistance.(23) These changes in sleep and sleep consolidation (uninterrupted sleep during the night) result from the interaction between neurophysiological maturation, behavioral self-regulation and environmental factors during infancy. A systematic review of more than three hundred scientific articles was conducted by a multidisciplinary

expert panel and recently published by the National Sleep Foundation.(24) The review comprises an update of age-related sleep duration recommendations (Figure 1):

- Newborns (0-3 months) are recommended to sleep 14-17 hours (11-13 and 18-19 hours of sleep may be appropriate to this age range, whereas less than 11 hours or more than 19 hours are not recommended);
- Infants (4-11 months) are recommended to sleep 12-15 hours (10-11 and 16-18 hours may be appropriate but less than 10 or more than 18 hours are not recommended);
- Toddlers (1 to 2 years) are recommended to sleep 11-14 hours (9-10 and 15-16 hours may be appropriate but less than 9 or more than 16 hours are not recommended).

Even so, there is significant variability in sleep needs from child to child and across age ranges.(25) As a result, there is no single “magic number” for the duration of sleep needed by children of a certain age, and recommendations are always based on a range of hours.(25) Some individuals might sleep longer or shorter than the recommended times with no adverse effects.(24) However, individuals with sleep durations outside the normal range may be engaging in volitional sleep restriction and are at risk for health problems.(24) The number of daytime naps follows developmental trends up to the age of two (a mean of 1.7±0.6 naps per day in 0-2 year-olds) and most children discontinue daytime napping between 3 and 5 years of age.(26) A cross-cultural study reported no difference in the number of naps across cultures, suggesting that this sleep measure has a more biological than cultural basis. (27)

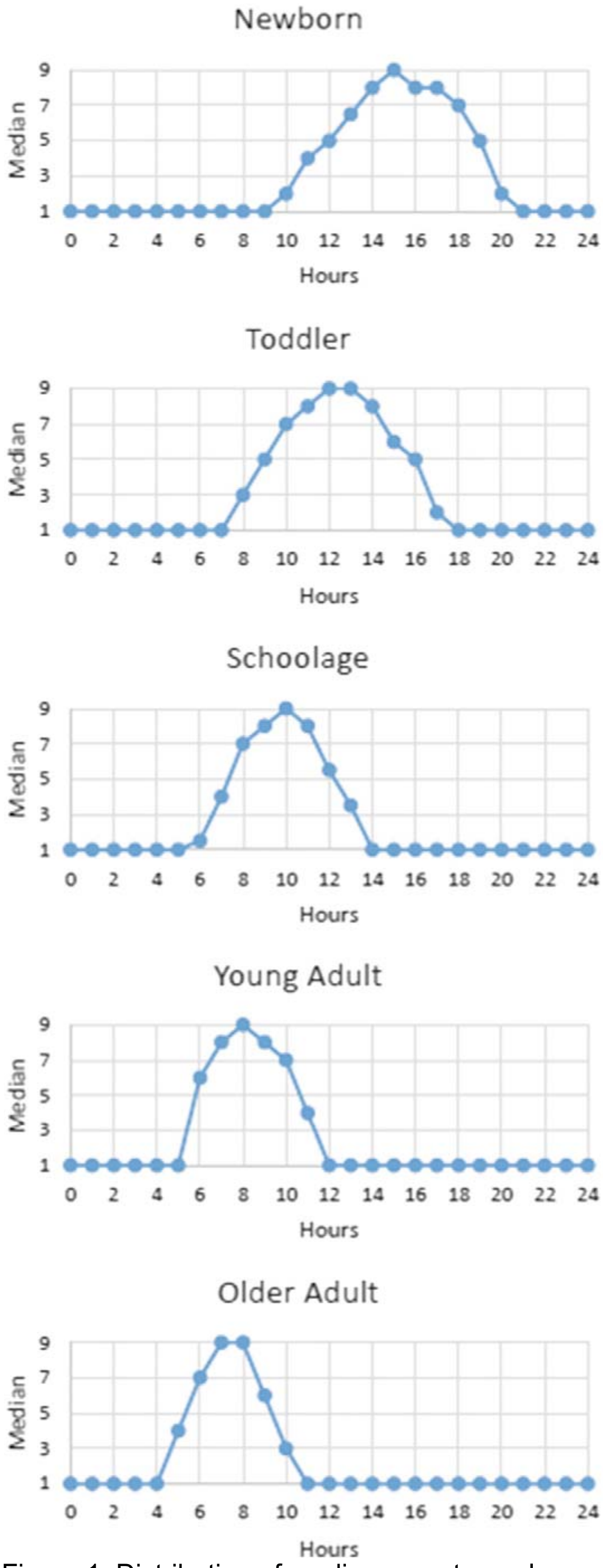

Figure 1. Distribution of median expert panel scores (extracted from Hirshkowitz M. et al, 2015)(24)

Henderson J et al (23), in a literature review including 26 articles, identified three operationally distinct aspects of sleep and sleep self-regulation during the first year of life: the longest sustained sleep period (LSP), the longest self-regulated sleep period (LSRSP) and sleeping through the night.

- The longest sustained sleep period (LSP) is a measure of the infant's physiological capacity for continuous sleep. The measurement period begins at the onset of any sleep episode and finishes at any signal of transition to full wakefulness. The measurement of LSP requires the use of objective assessments of infant sleep such as videosomnography, polysomnography and actigraphy, as subjective measures such as parental diaries and sleep questionnaires cannot distinguish sleep from quiet wakefulness, and cannot robustly quantify the duration of the LSP. The greatest rate of change in LSP has been found to occur between the first and the second month of life. There is a plateau between three and twelve months when LSP ranges from 4.72 to 6.71 hours. The average LSP (and standard deviation) at 1, 2, 3, 6 and 12 months is 3.57±1.21; 5.16±1.58; 5.54±1.5; 5.84±2.25; and 5.74±1.98 hours, respectively. This evidence suggests that the physiological ability for continuous sleep develops rapidly over the first three months after which it remains relatively stable.

- Longest self-regulated sleep period (LSRSP) is the longest uninterrupted period of behavioral quiescence that the infant is able to maintain. It comprises episodes that include periods of uninterrupted sleep, quiet wakefulness and re-initiation of sleep without parental intervention, with any one such episode terminating when infant indicates so by crying or calling out. The LSRSP may be measured either by videosomnography or by parental reports. Videosomnography measures periods of sleep, quite wakefulness and signaling. Parent maintained sleep diaries record the time and duration of each night awakening (complete arousal, excluding rapid-eye-movement sleep noises or self-soothing) that lasts more than 2 minutes.

There are three developmental trends in LSRSP across the first year of life: between 1-4 months when the greatest change in LSRSP duration occurs (particularly between the first two months); between 4-8 months, when there is a plateau; and between 9-12 months, when there is a small but steady increase in the mean LSRSP.(23) Infants experience a mean LSRSP of 6.98±1.96 hours at one month of age, increasing to 7.41±1.93 hours at two months, followed by 8.58±2.05 hours

at three months of age, 9.70±1.98 hours at six months of age, and 10.25±1.90 hours at twelve months of age.

- Sleeping through the night (STN) is a measure of the infants' ability for sustaining uninterrupted sleep during a predetermined nocturnal period. Three criteria are commonly used to assess STN: (i) uninterrupted sleep from 24:00 to 05:00 hours, (ii) between 22:00 and 06:00 hours, (iii) 8 hours of uninterrupted sleep between onset and awakening; all take into account the number of consecutive nights and weeks over which the STN is assessed and requires 5 of 6 nights of STN by any of the criteria above to be classified as STN. In the review by Henderson J et al,(23) in a majority of the studies, most of the 3-month old infants (56-71%) sleep through the night. This prevalence increases with age and by 12 months of age, 80-95% of infants are reported to sleep through the night.

These are three substantially different aspects of infants' sleep that are important and informative. The LSP informs us about the infants' physiological ability to sleep and is a measure of their neurological maturation; the LSRSP, includes the SLP but includes the infants' ability to independently reinitiate sleep after an awakening; and the STN is the aspect that carries most social validity as it is likely to socially approximate the parents' sleep.(23) The LSP development does not vary across time or population, having a strong biological determination, whereas LSRSP and STN are much more influenced by environmental factors.(23)

In The Longitudinal Study of Australian Children, a population-based birth cohort that followed-up 2926 children aged 0-1 to 6-7 years, Magee CA et al. investigated the presence, nature and consequences of sleeping duration trajectories over physical, emotional and social health-related quality of life.(21) The authors identified four distinct patterns of sleep duration during childhood: typical sleepers (40.6%), initially short sleepers (45.2%), persistent short sleepers (11.6%), and poor sleepers (2.5%), with differences in sleep duration between trajectories being more pronounced in the first two years of life (see Figure 1 below extracted from the paper by Magee et al.)(21) Sleep duration for poor sleepers was on average 4:40 hours and 1:41 hours less than those of typical sleepers at 0-1 and 2-3 years of age, respectively. Persistent short sleepers had sleep duration 0:20 to 1:09 hours less than typical sleepers across all ages, and the initially short sleepers presented in average 1:52 hours less of sleep at 0 to 1 year of age. In comparison to typical sleepers, initially short sleepers, persistent short

sleepers and poor sleepers presented high rates of sleep problems and irritability. Persistent short sleepers had lower physical (ß=-0.17; p=0.005), emotional (ß=-0.20; p<0.001) and social (ß=-0.16; p=0.006) health-related quality of life in comparison to typical sleepers. Initially short sleepers (ß=-0.13; p=0.004) and poor sleepers (ß=-0.35; p=0.003) had poorer physical health related quality of life.

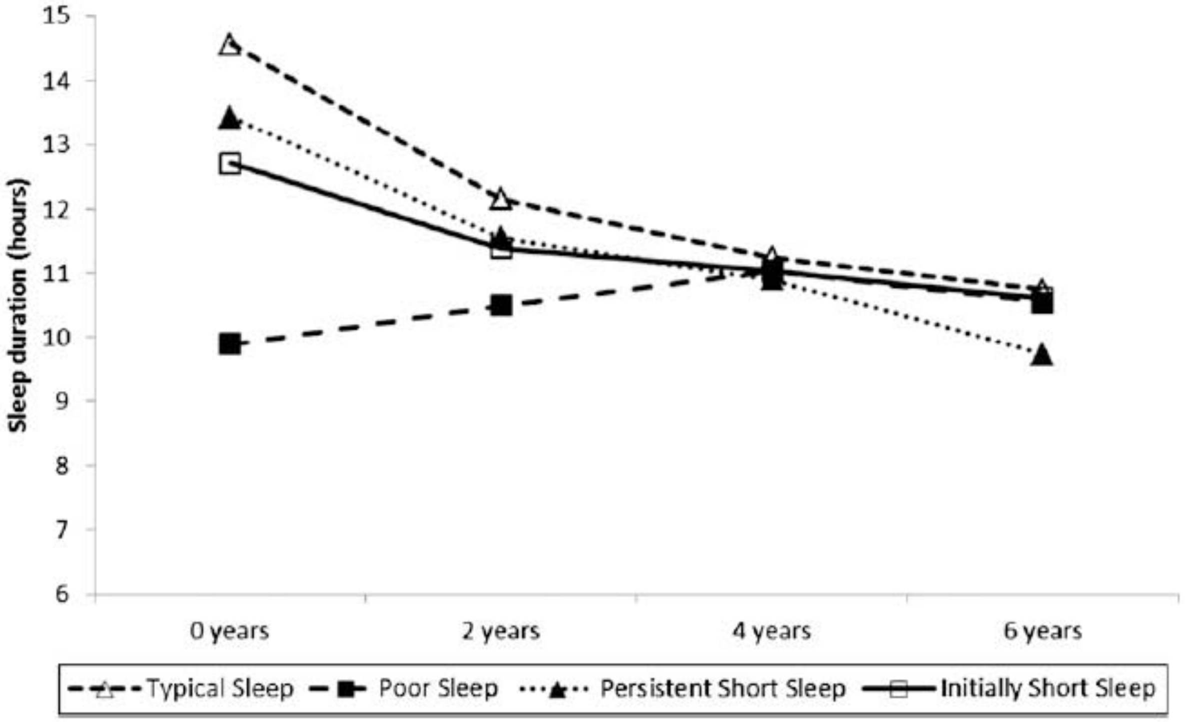

FIGURE 1
Average sleep durations for each of the 4 sleep trajectories in this sample of Australian children followed from age 0 to 1 years to 6 to 7 years.

Figure 2. Extracted from Magee CA et al., 2014(21)

2) SLEEP AND DEVELOPMENT IN CHILDHOOD

The sleep–wake system undergoes a dramatic process of maturational change during the first few months of life.(28) However, this may be affected by the individual characteristics of infants and children. The average newborn spends 16 hours in sleep, distributed in 5 to 6 episodes during 24 hours. By 6 months of age (or even earlier), most infants attain one prolonged and relatively consolidated sleep episode during the nighttime hours and most wakefulness occurs during the

daytime hours. This process is related to brain maturation and melatonin secretion by the pineal body in relation to the environmental light–dark cycle (melatonin is secreted during dark hours and eliminated by light); and is closely linked to changing sleep–wake patterns throughout life.(29, 30)

The role of sleep in learning, memory, behavior and other neurobiobehavioral functions has been extensively studied.(31) Many studies have demonstrated that sleep problems and sleep fragmentation are associated with an increased risk of learning difficulties and compromised neurobehavioral functioning in children.(31) However, there are only a small number of studies that have experimentally tested the effects of sleep restriction and extension in affected children.(31) Two key limitations of these study designs are (i) all studies were laboratory-based and not necessarily reflective of the child's sleeping environment at home and (ii) the results were presented based upon a single night of drastic sleep curtailment which does not take into account the cumulative effects of mild-moderate sleep curtailment.(28, 32)

Seventy-seven children were recruited from regular classes in two distinct age groups: fourth grade (N= 42, mean age: 9.80±0.64 years) and sixth grade (N= 35; mean age: 11.58± 0.50 years) to participate in an experimental study.(33) Each child completed the study according to a 6-day protocol. On Day 1, each child received a package that included one actigraph and daily reports that were used to assess sleep and related parameters over the 6-day period. During Days 1 and 2, the child was instructed to sleep as he or she regularly sleeps. In the morning of Day 1 or 2 (between 8:00 a.m. and 10:00 a.m.) the child's baseline neurobehavioral functioning was tested. In the afternoon of Day 3, the children and their parents received a phone call indicating to them how to modify the child's sleep schedule for the following 3 days. According to a random assignment, 40 children were asked to go to sleep 1 hour earlier and 37 were asked to go to sleep 1 hour later than their regular bedtime. The neurobehavioral functioning was tested twice (baseline and post-intervention) with six age-appropriate tests to assess the following domains: motor speed, vigilance and motor reaction, sustained visual attention, response inhibition, visual memory, visual scanning, visual-motor speed, working memory, attention, working memory, and learning strategies. The children who extended their sleep significantly improved their performance on the digit forward memory test, whereas the performance of the other children did not change. Children in the sleep-extension group significantly improved their reaction

time, whereas the performance of the other children did not change significantly. On the simple reaction time test, performance of the children from the sleep-restriction group and of children who failed to extend or shorten their sleep by at least 30 min significantly deteriorated whereas performance of the children from the sleep-extension group remained stable.

To investigate whether two common sleep quality parameters “long wake episodes” (number of wake episodes ≥5 min) and “sleep efficiency” (defined as % of epochs scored as sleep between sleep onset and offset) could modify the relation between children’ s intelligence and academic achievement, Erath S. A. et al.(3) conducted a cross-sectional study comprising 280 fourth- and fifth-graders aged 8 to 10 years, from public schools in the United States. Sleep was assessed during seven consecutive nights of actigraphy. Children’ s performance on standardized tests of intelligence and academic achievement were obtained. Age, sex, ethnicity, income-to-needs ratio, single parent status, standardized body mass index, chronic illness and pubertal development were controlled in analyses. Higher intelligence was associated with higher academic achievement among children who exhibited fewer and more long wake episodes (ß = 2.69, SE = 0.20, $P < 0.001$; ß = 1.92, SE = 0.20, $p < 0.001$, respectively), and among children who exhibited higher and lower sleep efficiency (ß = 2.55, SE = 0.14, $p < 0.001$; ß = 2.06, SE = 0.14, $p < 0.001$, respectively). However, the association between intelligence and achievement was slightly weaker among children with more long wake episodes or lower sleep efficiency.

A cohort of sixty-two healthy born full-term children and their parents were recruited from the Massachusetts site of the National Institute of Child Health and Human Development Study of Early Child Care to participate in a study which investigated associations between children's sleep and wakefulness at 7, 19 and 31 months and cognitive at 24 months and language skills at 36 months outcomes. Parents completed at least four days of sleep interview data at each of the three time points resulting in a total of 12 days of sleep data. Dearing E. et al.(34) showed that after controlling for characteristics of children and mothers (as infant temperament and mother's psychological well-being), children whose sleep was more rhythmic at 7 and 19 months had higher mental developmental scores (measured by Bayley Scales) at 24 months and improved language skills at 36 months (measured by Reynell Developmental Language Scales).

To investigate whether infants with more regular sleep/wake and longer sleep durations patterns (as measured with the use of both actigraphy and parental sleep diary) would have an easier temperament and higher developmental scores, Spruyt K et al.(35) followed twenty healthy term infants at monthly intervals over the first year of life. Temperament was assessed using the Early Infant Temperament Questionnaire (EITQ) at 3 months and the Revised Infant Temperament Questionnaire (RITQ) at 6 and 11 months. Mental, motor and behavioral development was assessed at 12 months using the Bayley Scales of Infant Development II (BSID-II). At all 3 ages studied increased nocturnal sleep was correlated with increased approachability. In addition, at 11 months, increased diurnal sleep duration was correlated with increased rhythmicity and adaptability.

Sher A et al.,(36) in a cross-sectional study with 10-month-old low-risk infants (N=50), found that a more fragmented sleep pattern and more motor activity in sleep were correlated with lower mental development scores (as measured by the BSID-II) when objective sleep characteristics were measured by actigraphy but not correlated with subjective maternal reports. None of the sleep measures were correlated with psychomotor developmental scores.

In a longitudinal study, Piteo AM et al.(37) investigated the influence of sleep duration and snoring (parent report) on developmental outcomes on the BSID-II in 6-month old infants. Infants aged between 0 and 3 months were recruited by the child and family health nurses during visits routinely conduced in South Australia. The final sample at 6 months consisted of 88 full term non-snoring infants and 16 infants who snored frequently from shortly after birth and continued to snore at 6 months. The analyses were adjusted to socio-economic status, gender, parental smoking, infant wheezing, reflux, eczema, breastfeeding patterns, nocturnal sleep duration, and number of nocturnal awakening per night. Cognitive ability at 6 months old was lower (mean = 95.3; SD= 4.3) in a screened group of snoring infants (children who began snoring frequently within the first month of life and who were still snoring) compared to control infants (mean = 100.6; SD= 3.9). Children who slept longer at night or had fewer nocturnal awakenings had higher social-emotional development scores than those who had disturbed or inadequate sleep (measured by Bayley Scales).

To test the hypothesis that sleep favors the development of higher cognitive functions requiring pre-frontal cortex involvement, Bernier A et al.(38) examined the concurrent and prospective link

between infant sleep regulation and subsequent executive function (EF). Sixty healthy full term infants and their mothers were recruited from birth lists provided by Canadian Ministry of Health and Social Services. Sleep regulation was assessed through a parent sleep diary at 12 and 18 months post-birth. Child EF was assessed at 18 months of age using a working memory task (search hidden objects). At 12 months, children's cognitive functioning was also assessed with mental development index of the BSID-II. The tasks conduced at 26 months evaluated three dimensions of EF: working memory, inhibitory control and set shifting. After controlling for socio-economic status as well as for child mental development (BSID-II scores), higher proportions of total sleep occurring at night time and fewer night awakenings (both at 12 and 18 months) were related to better performance up to 14 months later on several EF tasks, especially those involving a strong impulse control component.

3) SLEEP AND GROWTH IN CHILDHOOD

Previous research has provided evidence for a relationship between sleep and growth in length/height. Secretory growth hormone (GH) bursts are known to rise after sleep onset during slow wave sleep with linear relationships between the amount of slow wave sleep and concomitant GH secretion.(39) However, to date, there are few published studies examining the associations between infant sleep, physical growth and weight gain.

The association between short sleep duration (fewer than 12 hours a day) at 6 to 24 months and being overweight at 36 months was demonstrated in a cohort study conducted by Taveras et al in the United States.(40) More recently, Tikotzky L et al,(10) in a cross-sectional study with 6-month old infants found an association between infant length and the sleep percentage at 6 months, after controlling for sex, birth weight, gestational age, and nocturnal breastfeeding. The sleep percentage explained an additional 5.4% of the variance in length ($ß = 0.16$, $p < 0.05$).

The association between sleep deprivation in the first years of life and overweight or obesity at 4 years of age was investigated among children from the 2004 Pelotas Birth Cohort in Brazil.(41) At the visits conducted at the 12, 24 and 48 months, the mother responded to questions regarding sleep and feeding habits, as well as socioeconomic and demographic characteristics of the family. Children who slept for less than 10 hours per night were considered sleep deprived. Children with a BMI Z-score 2

standard deviations above limits established by the World Health Organization were considered overweight, and those with a BMI Z-score above 3 standard deviations were considered obese. Of the 4263 live births in Pelotas in 2004, 4231 were enrolled in the study. Of those, 94%, 93.5% and 92% were re-evaluated on the 12, 24 and 48-month follow-ups, respectively. Sleep-deprived children at any follow-up between 0-48 months had a 33% higher chance of overweight/obesity at 48 months of age (prevalence ratio 1.33; 95%CI 1.03-1.71). This association was maintained after adjusting for possible confounders.

Lampl M. et al.,(8) in a study to test the hypothesis that sleep and episodic (saltatory) growth in infant length are temporally coupled processes, continuously recorded sleep onset and awakening for 23 infants over 4-17 months (n = 5798 daily records) by means of daily parental diaries. Growth measurement protocols included weekly, semi-weekly, or daily assessments. The authors found that individual saltatory growth in body length was significantly associated with increases in both total daily sleep hours and the number of sleep bouts. This was a nonlinear relationship among individuals, with episodic, aperiodic, pulsatile increases in sleep non-randomly concordant with saltatory length growth within 2 days.

Jiang Y et al., in a cross-sectional study carried out with 143 lean scholar children aged 10-11 years, in Shangai, found that lean children (defined as children's body mass index < 15th percentile, according to domestic data) improved height by sleeping ≥10 hours per day (ß = 0.57, p = 0.011).(42)

However, reports examining the association between sleep physiology and growth hormone secretion are conflicting, and while some studies provide evidence of such a relationship (9, 43-45) others have not found an association between parent-reported sleep duration and child growth.(46-48)

4) INTERVENTIONS TO IMPROVE SLEEP QUALITY IN INFANCY

- Types of interventions

Behavioral interventions have increasingly been recognized as a first-line treatment of choice for infant and toddler sleep disturbances, which includes difficulty settling or night waking.(49, 50) The term ‘‘sleep hygiene’’ encompasses (i) changes in sleep environment to promote good quality sleep, (ii) engaging the child and their parents in routines and practices that encourage sleep of good quality and

sufficient duration and (iii) the practice of soothing activities during wakefulness aiming to propitiate sleep onset.(51) The commonly used activities that are promoted via sleep hygiene include having a consistent bedtime and wake-up time for both nocturnal and daytime sleep (among children at the age group where naps are considered physiological); establishing an appropriate place to initiate sleep; and avoiding environmental and behavioral associations that may delay or interrupt sleep onset (being rocked to sleep, parents laying on the child's bed until sleep onset, nursing to sleep, watching TV in bed, or drinking beverages rich in caffeine around bedtime).(52)

Systematic reviews of the literature on behavioral interventions to promote sleep hygiene identified six approaches that have been used: (5, 49)

- Extinction: Parents should put the child in bed at a pre-specified time and ignore the child until a certain time on the following morning. During this period, parents monitor children to avoid the possibility of injury. The method is based on eliminating the acts that reinforce certain behaviors (such as crying on awakening), aiming at their extinction over time. The greatest difficulty in implementing this strategy is the parents' lack of consistency and the technique may be anxiety-promoting for parents.

- Gradual extinction: This technique consists of ignoring the demands of the child for specific time periods. These periods are usually determined by the child's age and temperament and the parents' discretion in relation to how long they tolerate their child's crying. Parents should calm the child for short periods, which usually range from 15 seconds to one minute. The technique aims to promote the child's capacity to self-soothe and return to sleep, without undesirable associations or parental interference.

- Minimal checking with systematic extinction: This is similar to the extinction method, however the child is checked on every 5 to 10 minutes, and the parent can comfort the child quickly when necessary, while monitoring for safety and illness.

- Programmed awakening: This consists of waking the child at night, 15 to 30 minutes before the usual time of spontaneous awakening, comforting the child and encouraging him/her to return to sleep. The number of programmed awakenings is a function of the number of spontaneous awakenings. The aim of the technique is that, over time, spontaneous awakenings are extinguished.

- Sleep remodeling: This consists of not allowing naps to occur at times that can disrupt nocturnal sleep onset, which comprises four hours before bedtime in children at an age range that allows two naps (up to 3 years of age) per day, and six hours before bedtime in children who usually have one nap a day.

- Positive routines: This method consists of the development of routines preceding bedtime that consist of peaceful and pleasurable activities. Another strategy that can be used is delaying the time to go to bed to ensure that, when lying down, the child falls asleep quickly, until the habit of falling asleep quickly is consolidated. Following this, the bedtime is anticipated and progressed towards in 15 to 30 minute intervals on successive nights until the bedtime considered appropriate is achieved.

The reviewed studies addressed a broad age range, varying from 3 months to 4 years, mostly comprising children in their first year of life. Mindell et al.'s 2006 systematic review of behavioral interventions for child sleep problems found that 49 of 52 programs led to clinically significant reductions in bedtime resistance and night waking 3 to 6 months later.(49)

- Randomized controlled trials that aimed to improve infant sleep

To evaluate the effectiveness of a behavioral-educational sleep intervention delivered in the early postpartum in improving maternal and infant sleep Stremler R et al.(53) conducted a randomized controlled trial at postpartum units of two hospitals in Canada. Participants were 246 primiparous women and their infants (n=123) or usual care (n=123) groups. The behavioral-educational sleep intervention included a 45-60 minute meeting with a nurse to discuss sleep information and strategies to promote maternal and infant sleep, a 20 page booklet with the content discussed, and phone contacts at one, two, and four weeks postpartum to reinforce information, provide support, and problem solve. The usual care group received calls at weeks one, two, and four to maintain contact without provision of advice. Primary outcome was maternal nocturnal (9 pm to 9 am) sleep (minutes) and secondary outcome was longest stretch of infant nocturnal sleep (minutes) measured at 6 and 12 weeks postpartum by actigraphy. Longitudinal mixed effects model analyses indicated no significant differences between the groups on any of the outcomes. The estimated mean difference in maternal

nocturnal sleep between the intervention and usual care groups was 5.97 minutes (95% confidence interval −7.55 to 19.5 minutes, p = 0.39).

Aiming to compare the effect of a behavioral sleep intervention with written information about normal sleep on infant sleep problems and maternal depression, Hiscock H & Wake M(54), between May 1998 and April 1999, carried out a randomized controlled trial nested within a larger survey in Well Child Clinics, in Melbourne, Australia. Participants were 156 mothers of infants aged 6-12 months with severe sleep problems according to the parents. The main outcome measures were maternal report of infant sleep problem and scores on Edinburgh Postnatal Depression Scale (EPDS) at two and four months. The intervention consisted in discussion on behavioral infant sleep intervention (controlled crying) delivered over three consultations. Mothers in the intervention group attended three private consultations, held fortnightly at their local maternal and child health center. The main intervention was controlled crying, whereby parents responded to their infant's cry at increasing time intervals, allowing the infant to fall asleep by itself. Mothers in the intervention group also received a sleep management plan, information about the development and management of sleep problems, and the same information about normal sleep patterns as the control group. They were asked to maintain daily sleep diaries until the first follow-up at 2 months later. Mothers in the control group were mailed a single sheet describing normal sleep patterns in infants aged 6 to 12 months based on Australian normative data. This sheet did not include advice on how to manage infant sleep problems. At two months more infant sleep problems had resolved in the intervention group than in the control group (53/76 versus 36/76; p = 0.005) and remaining sleep problems were less severe in the intervention group (p = 0.01). Overall depression scores fell further in the intervention group than in the control group (mean change -3.7, 95% confidence interval -4.7 to -2.7, versus -2.5, -1.7 to -3.4; p = 0.06). By four months, however, changes in sleep problems and depression scores were similar.

In 2003, Hiscock H et al.(55) replicated that intervention(54) in a cluster randomized trial within the existing universally available, state-wide primary health care service (49 Maternal and Child Health centers in Melbourne, Australia), by training the well-child care providers themselves to manage infant sleep problems in families from a broad socio-demographic sample. At the first consultation (8-month well-child visit) nurses elicited the nature of the sleep problem, identified solutions, and wrote an

individualized sleep management plan with the mother. Two handouts discussed normal sleep patterns at 6–12 months and sleep associations and their causal role in sleep problems. Handouts on managing problem overnight feeding (reducing volume/time spent feeding over a week) and dummies (removal or teaching infant to replace own dummy) were also available. Participants were 328 mothers reporting an infant sleep problem at 7 months. Maternal report of infant sleep problem, depression symptoms by means of the EPDS when infants were 10 and 12 months old were the main outcome measures. Prevalence of infant sleep problems was lower in the intervention than in control group at 10 months (56% versus 68%; adjusted OR 0.58; 95% CI: 0.36 to 0.94) and 12 months (39% versus 55%; adjusted OR 0.50; 0.31 to 0.80). EPDS scores indicated less depression at 10 months (adjusted mean difference -1.4; -2.3 to -0.4) and 12 months (-1.7; -2.6 to -0.7). At 2 years (17 months after random assignment), mothers in the intervention group were less likely than control mothers to report clinical depression symptoms: 15.4% versus 26.4% (EPDS community cut point >9) and 4.2% versus 13.2% (EPDS clinical cut point >12).(56) Neither parenting style nor child mental health differed markedly between the intervention and control groups. A total of 27.3% of children in the intervention group versus 32.6% of control children had a sleep problem. In a 5-year follow-up (when children were 6 years old), there were no differences between intervention and control families for children's emotional (p = 0.8) and conduct behavior scores (p = 0.6), sleep problems (9% versus 7%, p = 02), sleep habits score (p = 0.4), parent- (p = 0.7) and child-reported (p = 0.8) psychosocial functioning, chronic stress (29% versus 22%, p = 0.4); child-parent closeness (p = 0.1) and conflict (p = 0.4), global relationship (p = 0.9), disinhibited attachment (p = 0.3); and parent depression, anxiety, and stress scores (p = 0.9) or authoritative parenting (63% versus 59%, p = 0.5), suggesting that in the long term, the group subject to behavioral sleep techniques did not differ from the control group.(57)

The intervention proposed and tested by Hiscock H.(54, 55) was replicated again between March 1, 2010, and June1, 2011, that time translated into a public health program and including infants at an earlier age (4 weeks). Caregivers of infants seen by their maternal and child health nurse at their first home visit (day 7–10 postpartum) in 4 local government areas in Melbourne, Australia, were invited to take part in a study to evaluate a prevention program for infant sleep and cry problems and postnatal depression.(58) The study was a randomized controlled trial with 781 infants born at 32 weeks or later

in 42 well-child centers. Follow-up occurred at infant age 4 and 6 months. The intervention included information about normal infant sleep and cry patterns, settling techniques, medical causes of crying and parent self-care, delivered via booklet and DVD (at infant age 4 weeks), telephone consultation (8 weeks), and parent group (13 weeks) versus well-child care. Outcomes included caregiver-reported infant night sleep problem (primary outcome), infant daytime sleep, cry and feeding problems, crying and sleep duration, caregiver depression symptoms, attendance at night wakings, and formula changes. There were no differences between groups in caregiver report of infant sleep, crying, or feeding problems at either 4 or 6-month follow-up. Compared to control caregivers, intervention caregivers at 6 months were less likely to score > 9 on the EPDS (7.9% versus 12.9%; adjusted odds ratio 0.57; 95% confidence interval 0.34 to 0.94), spend > 20 minutes attending infant wakings (41% versus 51%, adjusted OR 0.66; 0.46 to 0.95), or change formula (13% versus 23%; $p < 0.05$).

Lee KA and Gay CL(59) conducted an intervention to evaluate the effectiveness of the Sleep Hygiene Intervention Package for Parents (SHIPP) in minimizing night-time arousals and promote sleep maintenance among mothers following the birth of their first child. The intervention consisted of three components: (a) mother–infant proximity (in many cultures this practice decreases the episodes and durations of nighttime arousals), (b) noise attenuation, and (c) low lighting. The intervention was evaluated in two cohorts with diverse demographic and socioeconomic backgrounds. Expectant couples were recruited between 2001 and 2003 from fee-based childbirth education classes at a large academic hospital in San Francisco, California (Sample 1) (75 mothers in intervention group and 77 in control group). Pregnant women were recruited between 2004 and 2008 from free prenatal classes and clinics serving low-income women (Sample 2) (102 mothers in intervention group and 50 in control group). The experimental intervention was introduced during the third trimester, before the birth of the infant and was presented to both parents in Sample 1 and only to the mother in Sample 2. Participants were provided with a laminated information card summarizing the components of the intervention, a bassinet (so that the infant could sleep beside the mother's bed), a white noise machine (to mask non-essential sounds, but did not prevent the mother from hearing the infant) and a night-light to use for nighttime infant care instead of brighter bedroom lighting. Participants randomly assigned to the control group were provided with a pamphlet containing information about how diet can influence sleep and

recommendations for healthy eating. Each participant was asked to wear a wrist actigraph for 48 hours as part of each assessment (at 1 and 3 months after the delivery of the intervention). In Sample 1, the intervention had no effect on the postpartum sleep of mothers or their partners. In Sample 2, the mothers in the intervention group had better postpartum sleep; they obtained more nocturnal sleep, had better sleep efficiency, and had less wake episodes after sleep onset than mothers in the control group. In *post hoc* analyses, the differences in maternal sleep were statistically significant only at 3 months postpartum.

Aiming to investigate the impact of a consistent bedtime routine on infant and toddler sleep and on maternal mood, Mindell JA et al.(60) randomly assigned 405 mothers and their infant or toddler (ages 7-18 months, n = 206; ages 18-36 months, n = 199) to a routine or control group. Only children whose mother reported that the child had a sleep problem (ranging from “small” to “severe”) were included. Children with an apparent significant sleep disorder (> 3 night wakings per night, awake > 60 minutes per night, or total daily sleep duration < 9 hours), current acute or chronic illness, or those who were routinely bathed before bed (after 16:00) ≥ 4 times per week (as a nightly bath was part of the bedtime routine in the study) were not eligible. In the first week of the study (baseline) mothers were instructed to follow their child’s usual bedtime routine. In the second and third weeks, mothers in the routine group were instructed to conduct a specific bedtime routine that included a bath (using a provided wash product), a massage (using a provided massage product), and quiet activities (e.g., cuddling, singing lullaby), with lights out within 30 minutes of the end of the bath. Mothers of toddlers were instructed to apply lotion (using a provided product) rather than massage. Mothers continued to put their child to bed as they normally did, whether they put their child to bed awake or stayed with their child until asleep (e.g., rocked to sleep). The only recommended change was the institution of the prescribed bedtime routine. Overall, children had decreased sleep onset latency, decreased number/duration of night awaking episodes, increased sleep continuity, and decreased maternal perception of sleep as a problem (P < 0.001). There were also decreases in parental report of number of times the child called out and number of times the child climbed out of the crib/bed, P < 0.001.

Symon BG et al.(61) conducted a randomized controlled trial to evaluate the impact of a single consultation with a trained registered nurse recommending behaviour-modification approaches to

improve sleep in newborns. Families were recruited from birth notifications published in the Adelaide Advertiser, the only South Australian daily newspaper, between October 1996 and March 1997. Parents and infants in the control group (n= 175) received usual care from their health providers. Parents in the intervention group (n= 171) were invited to attend a 45-minute consultation with a trained research nurse when their infant was 2–3 weeks old. The consultation included a tutorial discussion and advice on normal sleep patterns in newborn infants. Each family was also given a 50-page book reinforcing the information. All participants were asked to record a sleep diary for 7 consecutive days when their child was aged 6 weeks and again at 12 weeks. The proportion of infants achieving a mean of 15 hours' sleep per 24 hours was significantly higher in the intervention group (65.6% at 6 and 57.4% at 12 weeks, versus 38.0% and 33.2% in control group, respectively), as were total duration of sleep, night sleep and daytime sleep per 24 hours in each follow-up assessment.

Over 9 months, women who delivered a live singleton baby of > 37 weeks gestation were approached in postnatal wards of a large general hospital and asked to participate in a randomized study comparing the effect of different parenting approaches on infant crying and sleeping(62). Of the 610 mothers who accepted to participate, 205 were assigned to the behavioral group, 202 to the educational group and 203 to the control group. The mothers were asked to complete one 24 hour diary at 1 week of infant age (baseline) and three successive 24 hour day diaries at each measurement point after randomization (3, 6, 9 and 12 weeks of age). At the age of 12 weeks, multilevel analysis showed that the odds of having a nighttime sleep bout of 5 hours or more were 2.6 times higher (95% CI 1.0–6.7) in infants in the behavioral group than in the control group when allowing for baseline measures, day-to-day variability and age.

Aiming to evaluate the efficacy of health education in reducing the incidence of sleep problems in infancy, Kerr SM et al.(63) in 1993 conducted an intervention involving 202 newborns in Glasgow, Scotland. The parents in the intervention group were visited once by the researcher at home when children were 3 months old. Written material in the form of a health education booklet was provided to parents from the intervention group. The advice was focused on settling methods and the importance of routine. The data relating to sleeping behavior were collected using a standardized interview. The sleeping behavior of the infants in both groups was compared when the children were 9 months old:

settling difficulties were more frequent among infants from the control group (39%) than in intervention group (21%) (p= 0.03); the median number of nights the control group woke was four, compared to two nights in intervention group (p= 0.04); and 46% of the control group woke two or more times per night, compared to 23% in intervention group (p= 0.02).

Lessons learned from those previous interventions indicate that methodological weaknesses to be avoided in future researches include: the use of parents' reports of infant sleep patterns obtained by direct interview or by using sleep diaries, rather than more valid reliable approach of an objective measure such as actigraphy; and the *a priori* assumption that parents have used the intervention if infant behavior changes in the desired way rather than to examine implementation directly.

IV) OBJECTIVES

1) MAIN OBJECTIVE

To examine the effect of a sleep hygiene based behavioral intervention on healthier sleep during the first year of life.

- SPECIFIC OBJECTIVE

1) To implement a randomized controlled trial to evaluate the effect of maternal counseling at 3 months post-partum over the child's auto-regulated nighttime sleep at ages 6, 12 and 24 months.

2) SECONDARY OBJECTIVES

1) To explore the effect of the intervention on physical growth at ages 12 and 24 months.

2) To explore the effect of the intervention over the neurocognitive development outcomes at ages 12 and 24 months.

3) To adapt and validate the INTERGROWTH-21st Neurodevelopment Assessment (INTER-NDA) for the assessment of cognition, motor skills, language, behavior, attention, and executive function in children at 12 months of age.

V) HYPOTHESES

1) Compared to children in the control group, children whose mothers received the sleep counseling at age 3 months:

- will have a longer average self-regulated nighttime sleep at ages 6, 12 and 24 months;
- will be taller at 12 and 24 months;
- will present a higher performance in the INTER-NDA-INTERGROWTH-21st instrument domains at age 12 and 24 months.

2) The INTER-NDA will show agreement with the Bayley Scales of Infant Development III edition (BSID-III) scores at 12 months and will show good test-retest and inter-rater reliability.

VI) METHODOLOGY

1) Study design

This will be a randomized controlled trial with children that belong to the 2015 Pelotas Birth Cohort.

2) Rationale for the study design

The random selection of participants into each arm, and the controlled way in which the trial will be carried out mean that all factors other than the intervention are considered equal. Therefore, randomized controlled trials are considered the gold standard design to the investigation of causal associations between interventions and outcomes. Although associations may be investigated under observational studies, causality cannot be inferred. Because this study aims to investigate the effect of a behavioral intervention to promote healthier sleep during the first year of life, a randomized controlled trial is the first choice design to achieve this objective.

3) Sample size

To calculate sample size, the following parameters were set: 95% confidence interval; double-sided statistical tests; 80% power; 1:1 ratio of intervention to control group; average self-regulated sleep period duration in the control group at 6 months as 9.70±1.98 hours,(23) 10.25±1.90 hours at 12 months,(23), and 10.30±1.26 hours at 24 months;(64) and self-regulated night sleep at least 30 minutes longer in the intervention group at 6, 12 and 24 months when compared to the control group.

The greatest sample size obtained was 251 children in each group. With an added 10% to account for losses to follow-up and for adjustments in the analyses the final number of children per arm is 276.

Table 1. Sample size needed to achieve 80% power to detect a mean difference of 30 minutes in self-regulated night sleep (SRNS) between intervention and control group.

Age (months)	SRNS in control group (hours)	SRNS 30 minutes longer in intervention group (hours)	N in control group	N in intervention group	Total N
6	9.70±1.98	10.12	251	251	502
12	10.25±1.90	10.75	174	174	348
24	10.30±1.26	10.80	98	98	196

4) Eligibility criteria

Three eligibility criteria will be employed to screen for the trial: to belong to the 2015 Pelotas Birth Cohort, to be born healthy from a single pregnancy at gestational age 37 weeks or greater, and to sleep on average less than 15 hours per 24 hours (daytime and nighttime sleep) at three months of age in the previous 2 weeks (as reported by the mother). The information on child sleep duration per 24 hours will be gathered during the interview with mothers from the entire cohort, by means of questions on the time the child is generally put in bed at night and the time the child generally awakes in the morning, as well as number and duration of daytime naps, number of wake episodes during the night and how long it takes to the child to fall sleep after a nocturnal awakening.

5) Outcomes definition

The main outcome will be the average nighttime self-regulated sleep duration (the maximum amount of time the child stays asleep or awake without awakening the parents),(23) at ages 6, 12 and 24 months. This outcome will be evaluated by using the actigraphy and diary records for 5 days before and after the intervention by the two groups. The Brief Infant Sleep Questionnaire (BISQ)(65) will be used at these three visits.

The secondary outcomes that will be measured are linear growth between age 3-12 and 12-24 months and neurocognitive development at 12 and 24 months. The effect of the intervention on linear growth will be estimated by comparing conditional growth between groups. At 3, 12 and 24 months of age anthropometric evaluation will include measures of length and weight. Length will be measured

using a foldable wooden infantometer, custom made for the study, using for measurement a nylon tape measure with 1 mm precision adhered to a groove carved into the body of the instrument. Mother and child weight will be measured using an electronic scale (150 kg capacity and 100 g precision), the mother being weighed first alone then holding the baby. The child's weight will be calculated as the difference between the two measures. The mother will be weighed clothed, but without heavy outfits, and clothes worn by the mother will be recorded. The child will be weighed undressed, whenever allowed by the mother. Otherwise, the child's clothing will be recorded.

The holistic neurodevelopment assessment designed and implemented by the Intergrowth-21st Project, and recently published (6) will be used to measure cognitive, motor, language, behavioral, attention, and executive function outcomes in children at 12 months. The 24 months version of the INTER-NDA has been validated against the BSID-III, and has been used in the neurodevelopment assessments of more than 1100 children from Brazil, India, Italy, Kenya, and the UK. The recently developed 12-month version of the instrument has not yet been validated and we propose to adapt and validate the instrument for this age group. The INTER-NDA12 was selected as the measure of choice for the assessment of neurodevelopment in the study because (i) it measures multiple dimensions of child development (ii) its administration time is 15 minutes (iii) it can be administered reliably and effectively by non-specialists (iv) it was designed to be suitable for use in low, middle and high income settings and in international populations and (v) it is based on objective reporting, rather than subjective judgment, of the child's performance.

6) Baseline assessment

During baseline (at 3 months of age), children in the intervention group will be compared to the control group in regards to their mean self-regulated nighttime sleep duration in the last 24 hours, measured by means of actigraphy and activity diary during 5 days. Mothers will also answer the Brief Infant Sleep Questionnaire (BISQ).(65)

As part of the 2015 Pelotas birth cohort protocols, information on a series of maternal, family and newborn characteristics (including socio-demographic status of the parents and household assets, weight, height and gestational age at birth, gender, older siblings, maternal depression at 3 month post-

delivery, breastfeeding patterns, feeding patterns at 3 months, and family and child sleep habits at age 3 months) will be collected at birth and at the 3-month visit follow-up.

7) Randomization

Randomization will be done in blocks of 6 children, at the study headquarters. For each child the interviewer will receive an opaque envelope, sealed, that will only be opened during the visit at day 5 post-consent. In this visit, the actigraphs and the diaries will be collected and the mothers randomized to the intervention group will receive the intervention. To measure adherence to the intervention, the mothers in the intervention group will receive an expanded version of the diary to be completed registering the number of nighttime awakenings, duration of nighttime awakenings, and measures taken to aid the child back to sleep. Those diaries will be completed during 45 days, starting at day 5 post-consent.

8) The intervention

The intervention will be delivered to the mothers at the child household. This will be a behavioral intervention composed of practices that promote self-regulated sleep habits,(66, 67) including information on:

- normal sleep behaviors during the first year of life;
- ideal conditions to promote sleep onset: environmental improvements that ensure restful sleep (no screen media, low noise and light);
- calming naptime routines and avoiding stimulating or stressing children just before naptime;
- practices that promote child self-regulation of sleep, including putting infants to sleep drowsy but awake; and on
- how to handle nighttime awakenings.(51)

A booklet with the intervention content to aid the mother in implementing the intervention will be used. A preliminary version of the booklet can be seen in Annex 1.

9) Instruments of data collection

- Questionnaires: baseline data on maternal, family and newborn characteristics are part of the 2015 Pelotas Birth Cohort protocols. Electronic questionnaires in notebooks or tablets will be used to undertake the interviews. At birth (perinatal evaluation), both the interview with the mother and newborn evaluation are currently being carried out within 24 hours of delivery. A standardized, pre-coded questionnaire composed of 13 sections is being applied to the mother: identification; delivery and newborn health; antenatal care and gestational morbidity; reproductive history; mother's characteristics and lifestyle; characteristics of work, father, and family income; tests taken by mother during antenatal care; newborn physical examination; and contact data (Annex 2).

The first follow-up (at age 3 months) started on April 1st, 2015. All children in the cohort will be sought for this follow-up visit. A seven-day window period was defined, including the day on which the child completed three months and the three days before and after this date. The structure of the 3-month follow-up questionnaire is similar to that of the perinatal questionnaire (Annex 3). An additional visit will be paid at 6 months to children enrolled in the trial.

These questionnaires will be used to collect information about parental characteristics: age, education, economic status, number of rooms at home, total number of persons living at home, number of siblings living at home; ante and perinatal information: antenatal care attendance, problems during pregnancy or delivery, type of delivery, gestational age, birth weight; infant weight and length at 3 months, feeding method (exclusive breastfeeding, predominant breastfeeding, partial breastfeeding, or formula), health problems (e.g. allergies, breathing problems), daycare setting (with mother at home, with babysitter or nursery); methods used as a means of soothing the infant for night and day (breastfeeding, bottle feeding, rocking); bed-sharing with the mother; and quality of the child sleep as perceived by the mother.

- Brief Infant Sleep Questionnaire (BISQ)(65): The BISQ questionnaire is a tool for screening sleep disorders in infants and toddlers (0-3 years). The instrument was developed and validated against sleep diary and actigraphic measures by Sadeh A (68) The translation to Brazilian Portuguese and the back-translation of the BISQ questionnaire were published by Nunes ML et al.(65) The questionnaire

variables include: nocturnal sleep duration (between the hours of 7pm and 7am); daytime sleep duration (between the hours of 7am and 7pm); number of night wakings; duration of wakefulness during the night hours (10pm to 6am); nocturnal sleep-onset time (the clock time at which the child falls asleep for the night); settling time (latency to falling asleep for the night); method of falling asleep; location of sleep; preferred body position; age of child; gender of child; birth order; and role of responder (who completed the BISQ). Completion of the BISQ requires 5 to 10 minutes. The parents will be instructed to refer to their child's sleep during the past week. Poor sleepers are defined as children who present one or more of the following criteria: wake more than 3 times per night; nocturnal wakefulness period greater than 1 hour; or total sleep time shorter than 9 hours. The BISQ questionnaire will be applied to the mother at the baseline interview and at the outcome evaluation visits at 6, 12 and 24 months of age.

- Actigraphy: at baseline (between the signature of the Informed Consent Form and the 5-day visit) the child will use an actigraph in the leg (even while bathing) to register the mean nighttime sleep. The children will wear the actigraph again during 5 days in each of the outcome visits (at the 6, 12 and 24 months of age). Actigraphy is an objective and validated method for assessing sleep–wake patterns in infants, children and adults. The actigraph is a wristwatch-like device that records body motion data that can be translated to reliable and valid sleep–wake measures. The actigraph will be attached to the infant's ankle. The Actigraphic Sleep Analysis program will be used to score the obtained data. It informs about nighttime awakenings and sleep duration; it is simple to use, without requirements for specialized professional, and is comfortable for the child to wear.(69, 70) The following sleep measures will be used: total night sleep period, from sleep onset time to morning awakening time; true sleep time, sleep time excluding all periods of wakefulness at night; number of night-wakings (lasting 5 min or longer).

- 5-day activity diary: in this diary mothers from intervention and control group will register the times of the day the child is asleep, awake, napping, and feeding (Annex 4). The diaries will be provided to the mothers just after their acceptance to participate in the study and before the opening of the child

allocation status (intervention or control group). Mothers from the two groups will complete those diaries for a 5-day period.

- 45-day activity diary: to promote and to measure the maternal adherence to the intervention, the mothers in the intervention group will receive an expanded version of the 5-day diary to be completed registering the number of nighttime awakenings, duration of nighttime awakenings, and measures taken to aid the child back to sleep. Those diaries will be completed during 45 days starting at day 5 post-consent. Only mothers from the intervention group will fulfill those diaries.

- Anthropometry: at 3, 12 and 24 months child length will be measured using a foldable wooden infantometer, custom made for the study. Mother and child weight will be measured using an electronic scale (150 kg capacity and 100 g precision), the mother being weighed first alone then holding the baby. The child's weight will be calculated as the difference between the two measures.

- **The INTERGROWTH-21st Neurodevelopment Assessment for 12 months and 24 months (INTER-NDA):** The INTER-NDA is a measure of cognition, motor skills (fine and gross motor), language (expressive and receptive), behavior, executive function, attention, and social-emotional reactivity for children aged 12 and 24 months. The 24-month version of the INTER-NDA covers the 22-30 age range and was designed and implemented by the INTERGROWTH-21st Project under which more than 1100 children in Brazil, India, Italy, Kenya, and the UK have been assessed. This 24-month version has been validated against the BSID-III scales, and has been evaluated for reliability, internal consistency and for administration by non-specialist field workers. The 12-month INTER-NDA is an extension of the 24-month-INTER-NDA to younger age ranges. It covers the 10-14 month age group. It is a 57-item measure consisting of a combination of directly administered, concurrently observed and maternally reported items. Both the 12 and 24 month versions of the INTER-NDA were designed to be international, population-based screening measures for early child neurodevelopment. They are based on objective reporting by (rather than the subjective judgement of) the examiner on the child's performance and aims to characterize child outcomes across a spectrum. The child's performance on

each item is reported on a 5-point scale. The INTER-NDA yields mean cognitive, language, motor, attention and social-emotional reactivity scores; as well as positive, negative and global behavior composites and sub-scale scores for receptive language, expressive language, fine motor skills and gross motor skills. Both versions of the INTER-NDA (i.e., at 12 and 24 months) were designed so as to be administered without the need for specialist personnel or infrastructure, with an administration time of 15-20 minutes.

10) Validation Study of the INTER-NDA for children aged 10-12 months

The INTER-NDA will be used to assess neurodevelopment in all children of the study at the 12- and 24-month assessments. As the INTER-NDA has not been validated for the 12-month age group, a sub-study nested within the larger study will be undertaken to validate the INTER-NDA for this age group. 100 children from the 2015 Pelotas Birth Cohort will participate in the validation study. None of these children would have been exposed to neuropsychological testing before. Children with known severe hearing or vision impairments will be excluded from participation as these may significantly confound cognition, language and behavioral scores. Twins will be excluded from participation as the normative profiles of brain growth and development in twins is not yet known. Children will be assessed on two consecutive days at 12 months ± 2 weeks of age. 50 children will be assessed for neurodevelopment on day 1 with the BSID-III and in the next day with the INTER-NDA. The remaining 50 children will be assessed using the INTER-NDA on Day 1, and the BSID-III on the following day. Hence, 50 children will be assessed using the INTER-NDA first and BSID-II second; and 50 children will be assessed using the BSID-III first and INTER-NDA second. Assessments will not be carried out on the same day in order to avoid fatigue and boredom spuriously contributing to differences in scores. The total and mean INTER-NDA scores for each subscale will be calculated, z-scored, and compared against the corresponding BSID-III subscales using four statistical methods, as recommended by Lee (71) and Bland & Altman (72): (1) Repeated measures *t*-tests in INTER-NDA and BSID-III scores between subjects; (2) Single measure intra-class correlation coefficients (ICCs) for absolute agreement between INTER-NDA and BSID-III scores; (3) Bias and limits of agreement statistics; and (4) The difference in INTER-NDA and BSID-III z scores will be plotted against the BSID-III score (Bland-Altman

plots) to identify whether the INTER-NDA scores differed systematically across different levels of the BSID-III.

A reliability assessment of the INTER-NDA will also be carried out. For each examiner, ten INTER-NDA and BSID-III assessments will be video-recorded. The same examiner will score these recordings 2 weeks after the initial assessment. The scores of the live and recorded assessment will be used to ascertain the test-re test reliability of the INTER-NDA and the BSID-III for each examiner using Cohen's kappa. The ten highest-quality video recordings will be selected and these will be watched and coded by all examiners and a Brazilian 'gold-standard' assessor simultaneously. The inter-rater and rater-gold standard reliability will be calculated using Cohen's kappa.

The data from the INTER-NDA assessments at 12 months will also be used to ascertain the internal consistency of the INTER-NDA in this age group using Cronbach's alphas. To evaluate the extent to which subscales on the INTER-NDA contribute to overall neurodevelopment at 12 month of age, the uni-dimensionality of the subscales will be evaluated using confirmatory factor analysis.

All INTER-NDA assessors will be trained following the INTERGROWTH-21st Project INTER-NDA training protocol by the expert trainer of the INTER-NDA.(6) The training includes elements of cultural customization of the INTER-NDA and translation of the measure to Brazilian Portuguese. The examiners will be selected so as to include at least one pediatrician and at least two non-specialists research assistants. This will enable an analysis of the degree of protocol adherence and reliability in scores between specialists and non-specialists.

11) Logistics of the trial

At the 3-month follow-up of the 2015 Pelotas Birth Cohort, children who (according to maternal report) sleep on average less than 13 hours per 24 hours (daytime and nighttime sleep) in the previous 2 weeks will be identified. A trained fieldworker will pay a visit to the mothers of those children at their households when they will be invited to participate in the study. Those who agree to participate will be presented with the informed Consent Form (CF) for signature and a following visit will be scheduled to within 5 days. Between CF signature and the 5-day visit, the child will use an actigraph device in the leg. The mother will be oriented on how to fill out a sleep diary registering the times of the day the child

is asleep, awake, napping, and feeding.

For each child enrolled in the study, the interviewer will receive an opaque envelope, sealed, that will only be opened at the 5-day visit to reveal the child allocation in the trial (intervention or control group). In this visit, the actigraphs and the 5-days diaries will be collected and the sleep recommendations will be delivered to the mothers from the intervention group.

Because this will be an efficacy trial mothers from the intervention group will receive a phone call on days 1 and 2 following the delivery of the intervention to check and support for possible maternal difficulties in implementing the recommended actions and to reinforce the content of the intervention. The same fieldworker who delivered the intervention will pay those phone calls. Mothers from the intervention group will receive a new diary to be fulfilled during the 45 days following the delivery of the intervention. This diary will collect information on the number of nighttime awakenings, duration of nighttime awakenings, and measures taken to aid the child back to sleep. To support mothers from intervention group on any infant sleep related issues that arise at any time after the delivery of the intervention, a telephone number to contact the study team will be provided.

The intervention group will subsequently receive five visits: at days 8 and 45 post CF signature and at 6, 12 and 24 months of age (see the trial flowchart in Figure 3). The visit at day 8 will have the objective of investigating maternal impressions about the child's sleep and to aid in managing difficulties implementing the intervention and in managing the child's sleep. The correct use of the 45-days diary will also be verified and reinforced during this visit. At this visit the mother will have the opportunity to solve any doubts about the implementation of the intervention and about the use of the diary. The visit on day 45 will have the objective of collecting the diaries and interviewing the mother about the child's sleep. At 6, 12 and 24 months of age, all study children (intervention and control group) will have measured for 5 days the duration of self-regulated nighttime sleep by means of actigraphy. At 12 and 24 months of age all study children (intervention and control group) will undergo anthropometry and neurodevelopment assessment. At the 5-day visit, the mothers of infants randomly allocated to the control group will be informed about the follow-up visits to be paid at ages 6, 12 and 24 months.

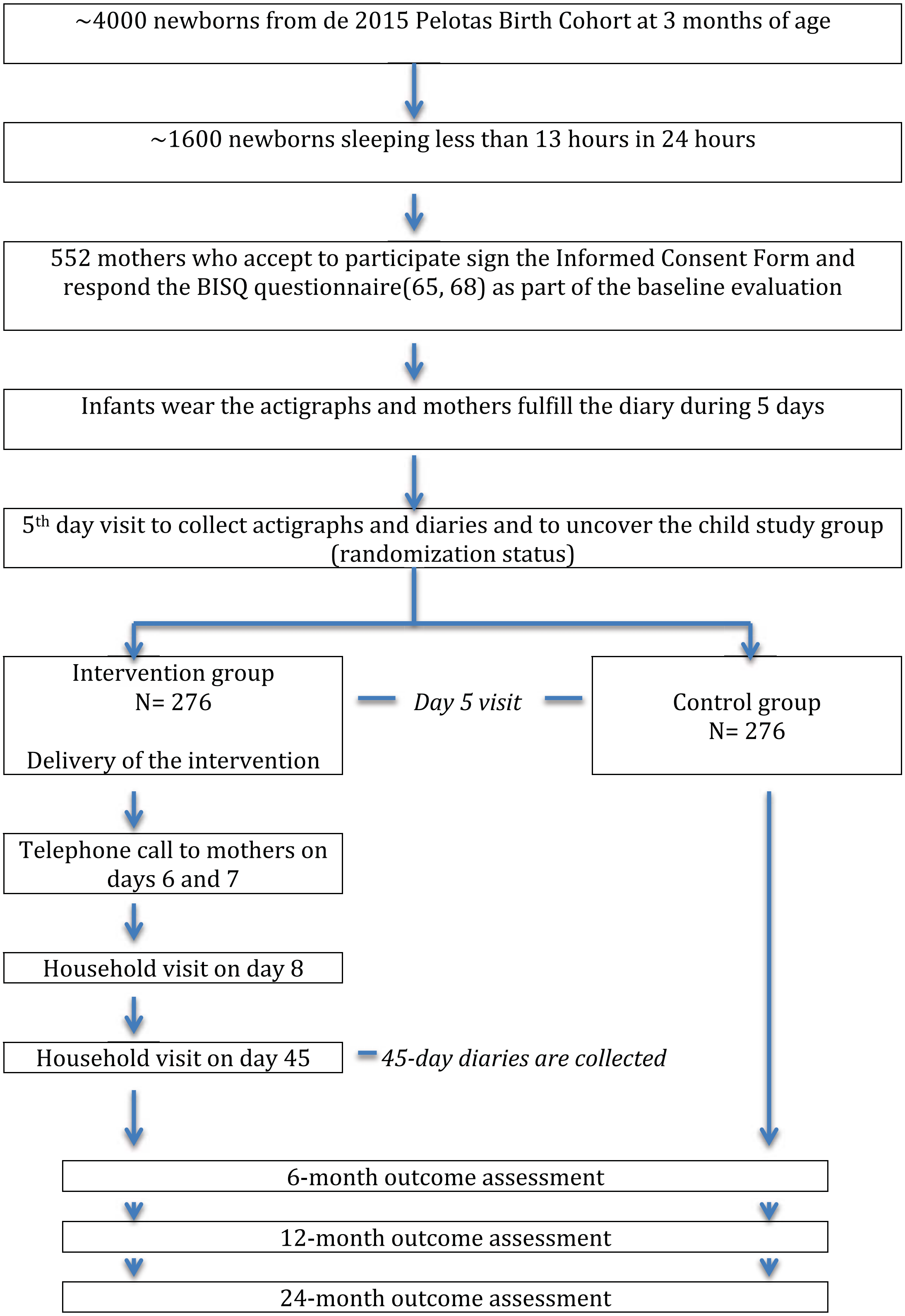

Figure 3. Trial flowchart

12) Study team

The fieldwork team will be composed of interviewers, fieldwork supervisors, and coordinators. All interviewers will have completed at least secondary education. Interviewer training will include general instructions in regard to the study protocol, reading of the questionnaire and fieldwork manual, detailed discussion of each question, and questionnaire administration among the trainees and later with mothers.

Special attention will be given to the training of fieldworkers on the delivery of the intervention. Written training material focusing on normal sleep behaviors, how to facilitate sleep onset and how to manage night awakenings will be produced to the study. Two experienced Pediatricians and a Psychologist will be the facilitator at the training sections. The training program will last 40 hours, with 40% of the time spent in practical sessions with mothers not belonging to the 2015 Pelotas Birth Cohort. The development of counseling skills will be emphasized. The performance of all participants will be formally assessed at the end of the training to document its effectiveness and to aid the coordinators in the selection of the fieldworkers.

Specific anthropometry techniques will be practiced at a daycare, until all interviewers are performing measurements correctly. About twice the number of interviewers necessary for each visit will be included in the training program. Interviewer selection will be based on a written test and performance evaluation during training.

13) Quality control

Quality control measures will include: use of pretested, standardized data collection forms; the construction of detailed interviewer guides; careful evaluation and selection of interviewers; thorough training on interviewing and anthropometric measurements, followed by standardization sessions with assessment of intra and inter-observer variability; and repetition by a supervisor of 5-10% of all interviews. Because this is an educational intervention strict blinding of interviewers and of the field supervisor regarding the intervention or control status of mothers and children will not be possible. However, as the outcome (self-regulated night sleep) will be assessed by means of an objective measure (actigraphy), the lack of blindness of the interviewers will not bias the results.

14) Pilot study

Prior to the study start, the sleep recommendations, the use of the actigraph and the fulfillment of the diaries will be tested in a pilot group of 20 mothers of 3-month old children. The aim of the pilot study will be to evaluate the acceptability of the recommendations and the feasibility of the study in the cultural context of child care practices in the city of Pelotas and to undertake the appropriate adjustments if necessary.

15) Methodology of analysis

The intervention and control groups will be compared in terms of baseline indicators collected at the perinatal and at the 3-month interview, including child sleep habits at age 3 months. Any differences between the two groups that had occurred despite the randomization will be adjusted for in the analyses. Analyses will be completed on an intention-to-treat basis. The Stata Statistical Software will be used in the analyses.

VIII) STRENGTHS AND LIMITATIONS OF THE STUDY

One of the strengths of this project is its feasibility. A population-based birth cohort study conducted in the same city in 2004 showed that an average of 300 children are born each month from mothers residing in the urban area of Pelotas at the city hospitals,(73) and that around 40% of them sleep less than 13 hours per 24 hours at age 3 months (data not published). Therefore, it is possible to obtain the necessary number of children during the year 2015 within the ongoing birth cohort study. To enable proper follow-up within the schedule of this call for proposals, children born between August, 1st and December, 31st, 2015 will be eligible to the trial.

This project is focused on sleep duration. The literature, especially observational studies, often does not distinguish between time in bed and actual sleep time. However, actual sleep time is typically less than time in bed, which biases data toward higher sleep duration estimates. By contrast, intervention studies using laboratory measured sleep time will typically produce shorter sleep durations. This study will employ maternal records (diaries) and actigraphy to measure and compare sleep habits

between intervention and control group before and after the delivery of the intervention. Those are reliable and recommended methods to assess the effect of interventions aiming to improve child sleep.(6)

Because this is an efficacy trial, maternal compliance is mandatory for the achievement of the study objectives. However, maternal adherence to the recommended practices is a potential limitation of this study. After the delivery of the recommendations, mothers from the intervention group will receive telephone calls in two separate days and will receive two additional home visits (at days 8 and 45 after the randomization) as strategies to promote compliance. The use of diaries to record the number of nighttime awakenings, duration of nighttime awakenings, and measures undertaken to aid the child back to sleep will help to measure maternal compliance with the study. The booklet containing key messages of the intervention will help the mother to remember the recommendations. Additionally, a telephone number to contact the study team will be provided to support mothers from the intervention group.

IX) ETHICAL ISSUES

The study protocol will be submitted to the Research Ethics Committee of the Medical School of the Universidade Federal de Pelotas, affiliated to the National Research Council. A written informed CF will be read to mothers so as to explain, in general terms, the aims and procedures of the study. In addition, the confidentiality of data, voluntary participation, and the possibility of leaving the study at any time without justification will be ensured. After clarifying any doubts the mother may have she will be invited to sign the term, keeping a copy for herself. The signed form will be archived at the study headquarters. A preliminary version of the CF to use with mothers from the pilot study can be seen in Annex 5.

X) PROGRESS INDICATORS

Table 2 outlines the chronogram of the activities necessary to successfully implement the project, monthly.

XI) EXPECTED RESULTS AND OPPORTUNITIES

It is expected that this study will demonstrate the beneficial effect of a sleep intervention on children's sleep behavior. This is a low cost intervention that does not require specialized professional to be delivered and that can be scaled-up to high-, middle- and low-income countries.

XII) SETTING AND ENVIRONMENT

The Centre for Epidemiologic Research at the Universidade Federal de Pelotas has adequate resources and facilities to conduct population-based studies as can be noted by the broad experience of the group in conducting cohort studies and intervention studies (www.epidemio-ufpel.org.br).

The principal investigator and the team of co-investigators have extensive experience in population-based studies, including RCTs. The group proposing this study coordinates the largest number of birth cohorts in low- and middle-income countries, with four birth cohort studies underway including over 15,000 individuals. The intellectual production of the principal investigator can be accessed here: http://lattes.cnpq.br/5322486498575710.

XIII) TECHNOLOGICAL AND SCIENTIFIC RELEVANCE

The negative impact of inadequate and insufficient sleep on children's physical and mental health are unquestionable, as well as its impact on cognitive function, academic performance and behavior, all of these being factors to which children in low- and middle-income countries are at higher risk.(25) Behavioral interventions targeting mothers and young children that can be delivered inexpensively and not requiring specialized training during the usual well-child visit in health care units can help prevent future issues by reducing the risk to which these children are exposed.

XIV) BUDGET in Brazilian Real and US Dollars (1USD = 2.99 REAL BRASIL in May 17, 2015)

Item	Total	
Scholarships	Brazilian Real	US Dollar
DTI-A scholarship (60 months) (2 scholarships for 30 months)	240 000	80 267.56
DTI-B scholarship (45 months) (3 scholarships for 15 months)	135 000	45 150.50
DTI-C scholarship (150 months) (10 scholarships for 15 months) (Trial)	165 000	55 183.95
DTI-C scholarship (12 months) (2 scholarships for 6 months) (Validation study)	13 200	4 414.72
**SUBTOTAL**	**553 200**	**185 016.73**
COSTS		
**THIRD PARTY SERVICES**		
Data collection system and instruments (programmer)	30 000	10 033.44
Transportation of field workers to household visits (~4370 return-tickets X $ 1.84)	24 035	8 040.80
Reimbursements	30 100	10 066.89
Booklet and activity-diaries final art and printing	30 000	10 033.44
Overhead for funding administration	23 920	8 000.00
**Other expenses**		
Air ticket (Toronto-Pelotas-Toronto)	4 500	1 505.02
Air ticket (Oxford-Pelotas-Oxford)	4 500	1 505.02
Living allowance (2 weeks x 2)	4760	1 591.97
Fieldworkers training (trial)	400	133.78
Training of assessors for the validation study	200	66.89
Pilot study	500	167.22
Office supplies	5 000	1 672.24
Other costs (including publication costs – open access)	20 000	6 688.96
**SUBTOTAL**	**177 915**	**59 505.67**
CAPITAL		
IT equipment (tablets; software for actigraphy)	25 000	8 361.20
Actigraph Motionwatch8 (40 units)	42 000	14 046.82
Bayley Kits (2)	6 240	2 186.96
INTER-NDA kit	938	313.40
Camcorder	10 000	3 344.48
**SUBTOTAL**	**84 178**	**28 252.86**
TOTAL	**815 293**	**272 775.26**

Budget justification:

Scholarships:

- DTI-A scholarships: 2 Research Assistants (PhD level) during 30 months will coordinate field work, data collection, data entry and will collaborate in data editing and analyses for the trial and for the validity study.
- DTI-B scholarships: 3 Research supervisors (Master level) will make telephone contact with the mothers, book household visits, apply the quality control questionnaires and take care of all organizational aspects of the two studies in a daily basis.
- DTI-C: 10 fieldworkers during 15 months will make the household visits (6 visits to mothers/children from the intervention group and 4 visits to mothers/children from the control group throughout the study) and will deliver the intervention to mothers from the intervention group (visit at Day 5).
- DTI-C: 2 fieldworkers during 6 months will apply the INTER-NDA and BSID-III instruments to children selected to the validation study (n= 100).

COSTS:
Third party services:
- Data collection system and instruments: the interviews will be recorded in tablets (1 per fieldworker). The tablets will be programmed to contain the structure of the questionnaire and the fields for anthropometry and neurodevelopment records. A programmer will prepare the virtual system at an estimated cost of $ 10 033.44.
- Transportation of fieldworkers to household visits: public rechargeable transportation cards will be provided to each fieldworker to reach the house of the participants of the trial and of the validation study totaling approximately 4370 return-tickets at $ 1.84 each ($ 8 040.80).
- Reimbursements to participants: by the end of the study, each of the participant mothers will receive the equivalent to $ 17.00 (seventeen USD) as reimbursement for the time spent with the study (totaling $ 10 068.89).
- Printing costs: a total of 280 booklets (containing the intervention messages) and 1300 activity diaries (to cover the baseline visit and the outcome visits at 6, 12 and 24 months of age, as well as the first 45 days following the delivery of the intervention) will be printed at an estimated total cost of $ 10 033.44.
- Overhead for funding administration: a 10% ($ 8 000.00) of the total costs of the project (excluding scholarships) was added as overhead for funding administration.

-
- **Other expenses:**
  - 2 Air tickets (Toronto-Pelotas-Toronto): to allow for one of the co-investigators (DGB) to attend 2 in-person meetings in Pelotas ($ 752.51 per travel) (total cost of $ 1 505.02).
  - 2 Air tickets (Heathrow-Pelotas-Heathrow): to allow for one of the co-investigator (MF) to attend 2 in-person meetings in Pelotas ($ 752.51 per travel) (total cost of $ 1 505.02).
  - Daily living expenses: 1 week per meeting in Pelotas for each of the two co-investigators (DGB and MF)
  - Fieldworkers training (trial study): 1 week (40 hours) training of 20 female candidates to fieldwork in interview techniques and in the delivery of the intervention, followed by the selection of the 10 with the best performance.
  - Training of assessors for the validation study: 1 week (40 hours) training of 4 female candidates in neurodevelopmental assessment, followed by the selection of the 2 with the best performance.
  - Pilot study: Pilot study to test the acceptability and feasibility of the intervention and to make the appropriate adjustments if needed will be carried on with a group of 20 mother/infant pairs, at an estimated cost of $ 167. 24.
  - Office supplies: cartridge for printer, paper, pens, envelopes, paper clips, post-it notes, file folders etc for the trial and for the validation study (total cost of $ 1672. 24).
  - Other costs: publication costs (open access) of papers reporting the methodology and the results of the trial and of the validation study (estimated total cost of $ 6 688.96).

CAPITAL:
- IT equipment: 12 tablets for data collection at a unitary price of $ 190.00; and the software ActiLife 6 for the actigraph data download and processing at an estimated price of $ 2244.00
- Actigraph Motionwatch8 (40 units): at a unit price of $ 351.17 (total of $ 14 046.82)

- Bayley-III test: 2 kits at a price of $ 1093.48 each, totaling $ 2 186.96.
- INTER-NDA kit: 2 kits at a price of $ 156.70 each (total of $ 313.40).
- Camcorder: a sub-sample of the INTER-NDA and BSID-III assessments will be video-recorded to assess the test-re test reliability of the two instruments. The camcorder price is estimated in $ 3344.48.

REFERENCES

1. Hoddinott J, Maluccio J, Behrman J, Flores R, R M. Effect of a nutrition intervention during early childhood on economic productivity in Guatemalan adults. Lancet. 2008;371(9610):411-6.
2. Victora CG, Adair L, Fall C, Hallal PC, Martorell R, Richter L, et al. Maternal and child undernutrition: consequences for adult health and human capital. Lancet. 2008 Jan 26;371(9609):340-57.
3. Erath SA, Tu KM, Buckhalt JA, El-Sheikh M. Associations between children's intelligence and academic achievement: the role of sleep. J Sleep Res. 2015 Feb 14.
4. Wilhelm I, Prehn-Kristensen A, Born J. Sleep-dependent memory consolidation--what can be learnt from children? Neurosci Biobehav Rev. 2012 Aug;36(7):1718-28.
5. Halal CS, Nunes ML. Education in children's sleep hygiene: which approaches are effective? A systematic review. J Pediatr (Rio J). 2014 Sep-Oct;90(5):449-56.
6. Fernandes M, Stein A, Newton CR, Cheikh-Ismail L, Kihara M, Wulff K, et al. The INTERGROWTH-21st Project Neurodevelopment Package: A Novel Method for the Multi-Dimensional Assessment of Neurodevelopment in Pre-School Age Children. PLoS ONE. 2014;9(11):e113360.
7. Fallone G, Acebo C, Seifer R, Carskadon MA. Experimental restriction of sleep opportunity in children: effects on teacher ratings. Sleep. 2005 Dec;28(12):1561-7.
8. Lampl M, Johnson ML. Infant growth in length follows prolonged sleep and increased naps. Sleep. 2011 May;34(5):641-50.
9. Takahashi Y, Kipnis DM, Daughaday WH. Growth hormone secretion during sleep. J Clin Invest. 1968 Sep;47(9):2079-90.
10. Tikotzky L, G DEM, Har-Toov J, Dollberg S, Bar-Haim Y, Sadeh A. Sleep and physical growth in infants during the first 6 months. J Sleep Res. 2010 Mar;19(1 Pt 1):103-10.
11. Taveras EM, Blackburn K, Gillman MW, Haines J, McDonald J, Price S, et al. First steps for mommy and me: a pilot intervention to improve nutrition and physical activity behaviors of postpartum mothers and their infants. Matern Child Health J. 2011 Nov;15(8):1217-27.
12. Paul IM, Savage JS, Anzman SL, Beiler JS, Marini ME, Stokes JL, et al. Preventing obesity during infancy: a pilot study. Obesity (Silver Spring). 2011 Feb;19(2):353-61.
13. Beebe DW. Cognitive, behavioral, and functional consequences of inadequate sleep in children and adolescents. Pediatr Clin North Am. 2011 Jun;58(3):649-65.
14. Kelly Y, Kelly J, Sacker A. Changes in bedtime schedules and behavioral difficulties in 7 year old children. Pediatrics. 2013 Nov;132(5):e1184-93.
15. Paavonen EJ, Porkka-Heiskanen T, Lahikainen AR. Sleep quality, duration and behavioral symptoms among 5-6-year-old children. Eur Child Adolesc Psychiatry. 2009 Dec;18(12):747-54.
16. Magee L, Hale L. Longitudinal associations between sleep duration and subsequent weight gain: a systematic review. Sleep Med Rev. 2012 Jun;16(3):231-41.
17. Patel SR, Hu FB. Short sleep duration and weight gain: a systematic review. Obesity (Silver Spring). 2008 Mar;16(3):643-53.
18. Chen X, Beydoun MA, Wang Y. Is sleep duration associated with childhood obesity? A systematic review and meta-analysis. Obesity (Silver Spring). 2008 Feb;16(2):265-74.
19. National Sleep Foundation. Children and Sleep.; Available from: http://sleepfoundation.org/sleep-topics/children-and-sleep.

20. Gaffney KF, Kitsantas P, Brito A, Kastello J. Baby steps in the prevention of childhood obesity: IOM guidelines for pediatric practice. J Pediatr Nurs. 2014 Mar-Apr;29(2):108-13.
21. Magee CA, Gordon R, Caputi P. Distinct developmental trends in sleep duration during early childhood. Pediatrics. 2014 Jun;133(6):e1561-7.
22. Santos IS, Mota DM, Matijasevich A. Epidemiology of co-sleeping and nighttime waking at 12 months in a birth cohort. J Pediatr (Rio J). 2008 Mar-Apr;84(2):114-22.
23. Henderson JM, France KG, Blampied NM. The consolidation of infants' nocturnal sleep across the first year of life. Sleep Med Rev. 2011 Aug;15(4):211-20.
24. Hirshkowitz M, Whiton K, Albert SM, Alessi C, Bruni O, DonCarlos L, et al. National Sleep Foundation's sleep time duration recommendations: methodology and results summary. Sleep Health. 2015;1:40-3.
25. Owens JA. A letter to the editor in defense of sleep recommendations. Pediatrics. 2012 May;129(5):987-8; author reply 9-91; discussion 8-9.
26. Galland BC, Taylor BJ, Elder DE, Herbison P. Normal sleep patterns in infants and children: a systematic review of observational studies. Sleep Med Rev. 2012 Jun;16(3):213-22.
27. Mindell JA, Sadeh A, Wiegand B, How TH, Goh DY. Cross-cultural differences in infant and toddler sleep. Sleep Med. 2010 Mar;11(3):274-80.
28. Sadeh A, El-Sheikh M. Xi. Sleep and development: conclusions and future directions. Monogr Soc Res Child Dev. 2015 Mar;80(1):177-81.
29. Crowley SJ, Acebo C, Fallone G, Carskadon MA. Estimating dim light melatonin onset (DLMO) phase in adolescents using summer or school-year sleep/wake schedules. Sleep. 2006 Dec;29(12):1632-41.
30. Sadeh A. Sleep and melatonin in infants: a preliminary study. Sleep. 1997 Mar;20(3):185-91.
31. Ednick M, Cohen AP, McPhail GL, Beebe D, Simakajornboon N, Amin RS. A review of the effects of sleep during the first year of life on cognitive, psychomotor, and temperament development. Sleep. 2009 Nov;32(11):1449-58.
32. El-Sheikh M, Sadeh A. I. Sleep and development: introduction to the monograph. Monogr Soc Res Child Dev. 2015 Mar;80(1):1-14.
33. Sadeh A, Gruber R, Raviv A. The effects of sleep restriction and extension on school-age children: what a difference an hour makes. Child Dev. 2003 Mar-Apr;74(2):444-55.
34. Dearing E, McCartney K, Marshall N, Warner R. Parental reports of children's sleep and wakefulness: longitudinal associations with cognitive and language outcomes. Infant Behavior and Development 2001;24(2):151-70.
35. Spruyt K, Aitken RJ, So K, Charlton M, Adamson TM, Horne RS. Relationship between sleep/wake patterns, temperament and overall development in term infants over the first year of life. Early Hum Dev. 2008 May;84(5):289-96.
36. Scher A. Infant sleep at 10 months of age as a window to cognitive development. Early Hum Dev. 2005 Mar;81(3):289-92.
37. Piteo AM, Kennedy JD, Roberts RM, Martin AJ, Nettelbeck T, Kohler MJ, et al. Snoring and cognitive development in infancy. Sleep Med. 2011 Dec;12(10):981-7.
38. Bernier A, Carlson SM, Bordeleau S, Carrier J. Relations between physiological and cognitive regulatory systems: infant sleep regulation and subsequent executive functioning. Child Dev. 2010 Nov-Dec;81(6):1739-52.
39. Van Cauter E, Plat L. Physiology of growth hormone secretion during sleep. J Pediatr. 1996 May;128(5 Pt 2):S32-7.
40. Taveras EM, Rifas-Shiman SL, Oken E, Gunderson EP, Gillman MW. Short sleep duration in infancy and risk of childhood overweight. Arch Pediatr Adolesc Med. 2008 Apr;162(4):305-11.
41. Halal C. Sleep deprivation and overweight/obesity among Brazilian children: Study from a

birth cohort. . Porto Alegre, RS, Brasil: Pontifícia Universidade Católica do Rio Grande do Sul (PUCRS). ; 2014.
42. Jiang YR, Spruyt K, Chen WJ, Shen XM, Jiang F. Somatic growth of lean children: the potential role of sleep. World J Pediatr. 2014 Aug;10(3):245-50.
43. Sassin JF, Parker DC, Mace JW, Gotlin RW, Johnson LC, Rossman LG. Human growth hormone release: relation to slow-wave sleep and sleep-walking cycles. Science. 1969 Aug 1;165(3892):513-5.
44. Finkelstein JW, Anders TF, Sacher EJ, Roffwarg HP, Hellman LD. Behavioral state, sleep stage and growth hormone levels in human infants. J Clin Endocrinol Metab. 1971 Mar;32(3):368-71.
45. Van Cauter E, Plat L, Copinschi G. Interrelations between sleep and the somatotropic axis. Sleep. 1998 Sep 15;21(6):553-66.
46. Gulliford MC, Price CE, Rona RJ, Chinn S. Sleep habits and height at ages 5 to 11. Arch Dis Child. 1990 Jan;65(1):119-22.
47. Knutson KL. The association between pubertal status and sleep duration and quality among a nationally representative sample of U. S. adolescents. Am J Hum Biol. 2005 Jul-Aug;17(4):418-24.
48. Jenni OG, Molinari L, Caflisch JA, Largo RH. Sleep duration from ages 1 to 10 years: variability and stability in comparison with growth. Pediatrics. 2007 Oct;120(4):e769-76.
49. Mindell JA, Kuhn B, Lewin DS, Meltzer LJ, Sadeh A. Behavioral treatment of bedtime problems and night wakings in infants and young children. Sleep. 2006 Oct;29(10):1263-76.
50. Ramchandani P, Wiggs L, Webb V, Stores G. A systematic review of treatments for settling problems and night waking in young children. BMJ. 2000 Jan 22;320(7229):209-13.
51. Gruber R, Cassoff J, Knauper B. Sleep health education in pediatric community settings: rationale and practical suggestions for incorporating healthy sleep education into pediatric practice. Pediatr Clin North Am. 2011 Jun;58(3):735-54.
52. Mindell JA, Meltzer LJ, Carskadon MA, Chervin RD. Developmental aspects of sleep hygiene: findings from the 2004 National Sleep Foundation Sleep in America Poll. Sleep Med. 2009 Aug;10(7):771-9.
53. Stremler R, Hodnett E, Kenton L, Lee K, Weiss S, Weston J, et al. Effect of behavioural-educational intervention on sleep for primiparous women and their infants in early postpartum: multisite randomised controlled trial. BMJ. 2013;346:f1164.
54. Hiscock H, Wake M. Randomised controlled trial of behavioural infant sleep intervention to improve infant sleep and maternal mood. BMJ. 2002 May 4;324(7345):1062-5.
55. Hiscock H, Bayer J, Gold L, Hampton A, Ukoumunne OC, Wake M. Improving infant sleep and maternal mental health: a cluster randomised trial. Arch Dis Child. 2007 Nov;92(11):952-8.
56. Hiscock H, Bayer JK, Hampton A, Ukoumunne OC, Wake M. Long-term mother and child mental health effects of a population-based infant sleep intervention: cluster-randomized, controlled trial. Pediatrics. 2008 Sep;122(3):e621-7.
57. Price AM, Wake M, Ukoumunne OC, Hiscock H. Five-year follow-up of harms and benefits of behavioral infant sleep intervention: randomized trial. Pediatrics. 2012 Oct;130(4):643-51.
58. Hiscock H, Cook F, Bayer J, Le HN, Mensah F, Cann W, et al. Preventing early infant sleep and crying problems and postnatal depression: a randomized trial. Pediatrics. 2014 Feb;133(2):e346-54.
59. Lee KA, Gay CL. Can modifications to the bedroom environment improve the sleep of new parents? Two randomized controlled trials. Res Nurs Health. 2011 Feb;34(1):7-19.
60. Mindell JA, Telofski LS, Wiegand B, Kurtz ES. A nightly bedtime routine: impact on sleep in young children and maternal mood. Sleep. 2009 May;32(5):599-606.

61. Symon BG, Marley JE, Martin AJ, Norman ER. Effect of a consultation teaching behaviour modification on sleep performance in infants: a randomised controlled trial. Med J Aust. 2005 Mar 7;182(5):215-8.
62. St James-Roberts I, Sleep J, Morris S, Owen C, Gillham P. Use of a behavioural programme in the first 3 months to prevent infant crying and sleeping problems. J Paediatr Child Health. 2001 Jun;37(3):289-97.
63. Kerr SM, Jowett SA, Smith LN. Preventing sleep problems in infants: a randomized controlled trial. J Adv Nurs. 1996 Nov;24(5):938-42.
64. Sadeh A, Mindell JA, Luedtke K, Wiegand B. Sleep and sleep ecology in the first 3 years: a web-based study. J Sleep Res. 2009 Mar;18(1):60-73.
65. Nunes ML, Kampff JPR, Sadeh A. BISQ Questionnaire for Infant Sleep Assessment: translation into Brazilian portuguese. Sleep Sci. 2012;5(3):89-91.
66. Douglas PS, Hill PS. Behavioral sleep interventions in the first six months of life do not improve outcomes for mothers or infants: a systematic review. J Dev Behav Pediatr. 2013 Sep;34(7):497-507.
67. Benjamin Neelon SE, Duffey K, Slining MM. Regulations to promote healthy sleep practices in child care. Pediatrics. 2014 Dec;134(6):1167-74.
68. Sadeh A. A brief screening questionnaire for infant sleep problems: validation and findings for an Internet sample. Pediatrics. 2004 Jun;113(6):e570-7.
69. So K, Adamson TM, Horne RS. The use of actigraphy for assessment of the development of sleep/wake patterns in infants during the first 12 months of life. J Sleep Res. 2007 Jun;16(2):181-7.
70. The International Fetal and Newborn Growth Consortium. INTERGROWTH-‐‑ 21st International Fetal and Newborn Growth Standards for the 21st Century: Sleep-wake cycle asessment manual. . Oxford: University of Oxford; 2014 [cited 2015 January 05, 2015]; Available from: http://www.medscinet.net/Intergrowth/patientinfodocs/Sleep-wake cycle assessment manual July 2014.pdf.
71. Lee J, Koh D, Ong C. Statistical evaluation of agreement between two methods for measuring a quantitative variable. Computers in biology and medicine. 1989;19(1):61-70.
72. Bland J, Altman D. A note on the use of the intraclass correlation coefficient in the evaluation of agreement between two methods of measurement. Computers in biology and medicine. 1990;20(5):337-40.
73. Santos IS, Barros AJ, Matijasevich A, Domingues MR, Barros FC, Victora CG. Cohort profile: the 2004 Pelotas (Brazil) birth cohort study. Int J Epidemiol. 2011 Dec;40(6):1461-8.

Table 2. Progress indicators

ACTIVITIES	YEAR 1 (2015)				YEAR 2 (2016)												YEAR 3 (2017)												YEAR 4 (2018)					
	MONTHS				MONTHS												MONTHS												MONTHS					
	S	O	N	D	J	F	M	A	M	J	J	A	S	O	N	D	J	F	M	A	M	J	J	A	S	O	N	D	J	F	M	A	M	J
Literature review	x	x	x	x	x	x	x	x	x	x	x	x	x	x	x	x	x	x	x	x	x	x	x	x	x	x	x	x	x	x	x	x	x	x
Instrument development	x	x																																
Intervention pilot		x																																
Training			x																															
Intervention			x	x	x	x	x																											
6 month outcomes						x	x	x	x	x																								
Validation of the INTER-NDA for 12 months	x	x	x	x	x	x	x	x	x	x	x																							
12 month outcomes												x	x	x	x	x																		
24 month outcomes																								x	x	x	x	x						
Data analyses and manuscript preparation																													x	x	x	x	x	x
