# Supplementary material for: Effect of Parental Counseling on Infants’ Healthy Sleep Habits in Brazil: A Randomized Clinical Trial
Source: JAMA Netw Open. 2019 Dec 20;2(12):e1918062. doi: 10.1001/jamanetworkopen.2019.18062 (PMC6991232; doi:10.1001/jamanetworkopen.2019.18062)
Supplement: Supplement 2. — eAppendix. Supplemental Methods eTable 1. Maternal Recall at 6 Months After Childbirth of the Advice Received at Baseline eTable 2. Effect of Intervention on Growth and Development at Age 12 mos eTable 3. Effect of Intervention on Growth and Development at Age 24 mos [file jamanetwopen-2-e1918062-s002.pdf]

Supplementary Online Content

Santos IS, Del-Ponte B, Tovo-Rodrigues L, et al. Effect of parental counseling on infants' healthy sleep habits in Brazil: a randomized clinical trial. *JAMA Netw Open*. 2019;2(12):e1918062. doi:10.1001/jamanetworkopen.2019.18062

eAppendix. Supplemental Methods
eTable 1. Maternal Recall at 6 Months After Childbirth of the Advice Received at Baseline
eTable 2. Effect of Intervention on Growth and Development at Age 12 mos
eTable 3. Effect of Intervention on Growth and Development at Age 24 mos

This supplementary material has been provided by the authors to give readers additional information about their work.

© 2019 Santos IS et al. *JAMA Network Open.*

eAppendix. Supplemental Methods

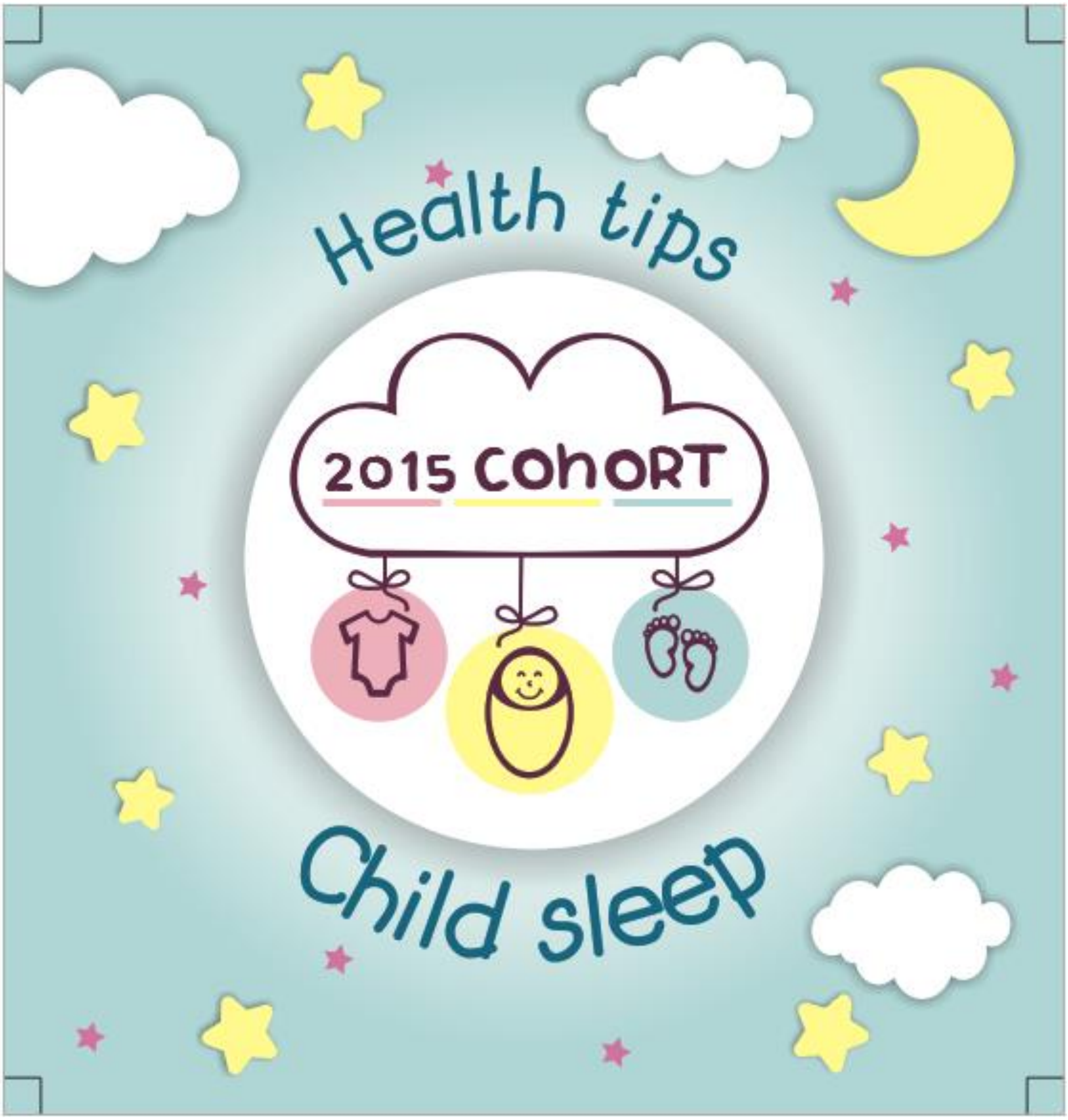

© 2019 Santos IS et al. *JAMA Network Open.*

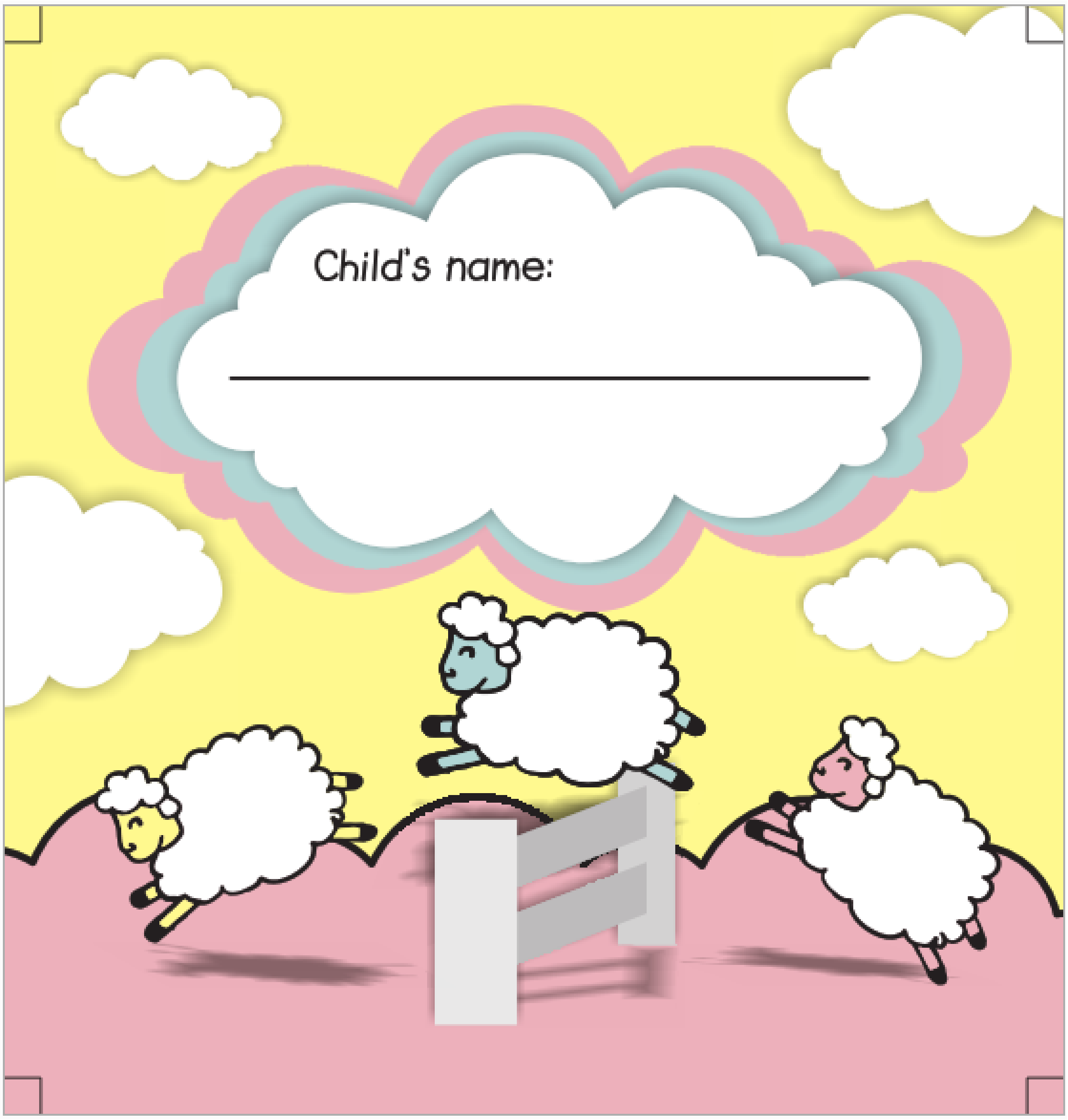

© 2019 Santos IS et al. *JAMA Network Open.*

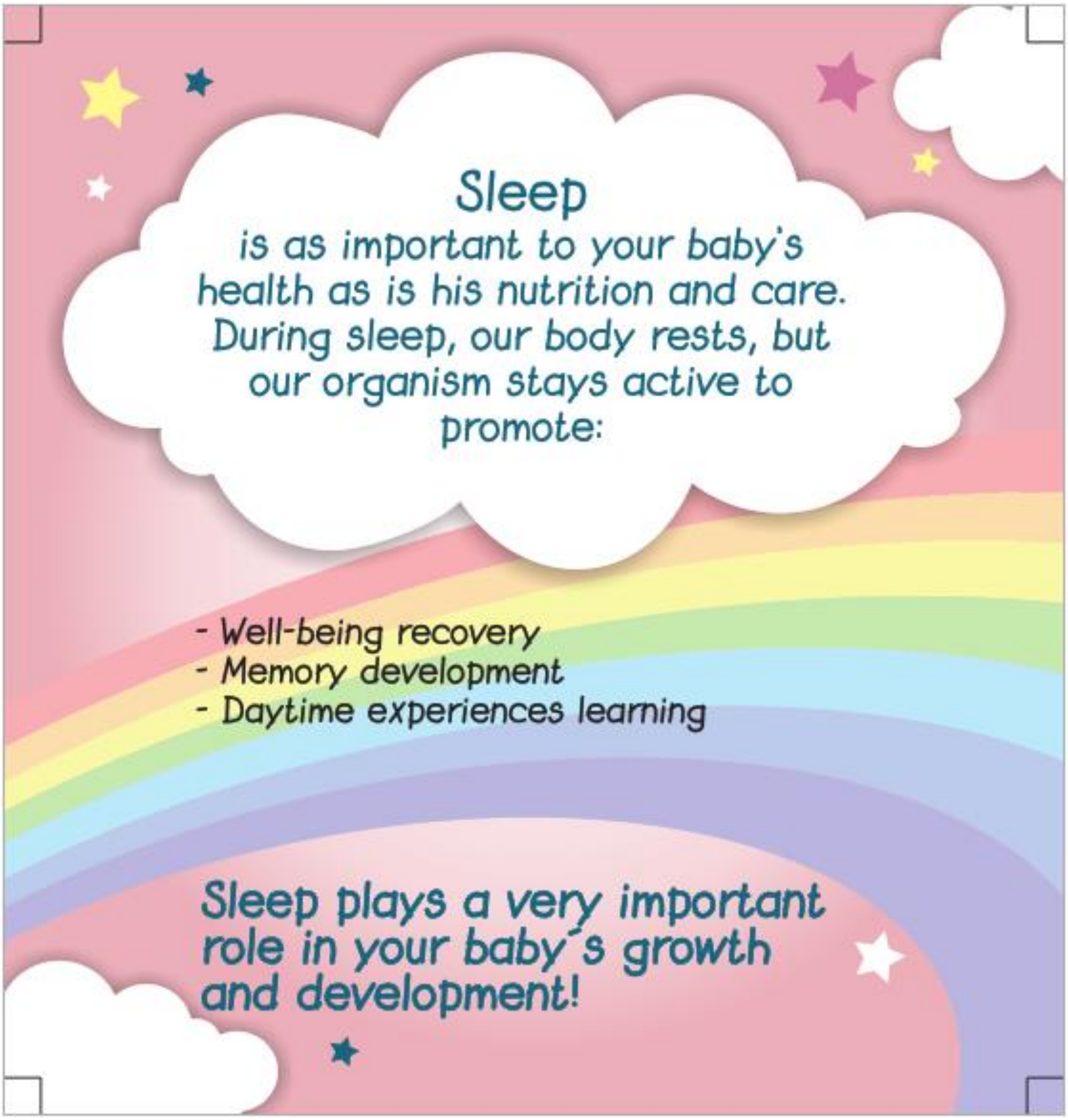

© 2019 Santos IS et al. *JAMA Network Open.*

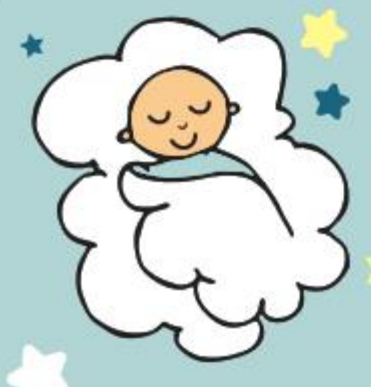

How much sleep does a baby need to grow and develop to his full potential?

From birth to age **3 months**, a baby needs approximately **14 to 17 hours** of sleep per day.

During this age, the baby will wake up frequently **(every 2 to 4 hours)** and his sleep is not yet organized as daytime sleep and night sleep.

From **6 months** of age onwards, sleep begins to get more organized with more hours of sleep at night and fewer hours during the day.

Nevertheless, babies continue to wake up at least twice during the night. The ideal total sleep times are based on the **24 hours** of the day, since at these ages daytime naps are a part of normal development.

Between **4 and 11 months** of age, total sleep time per day should be between 12 and 15 hours.

From **1 to 2 years** of age, children should sleep between **11 and 14 hours** a day.

© 2019 Santos IS et al. *JAMA Network Open.*

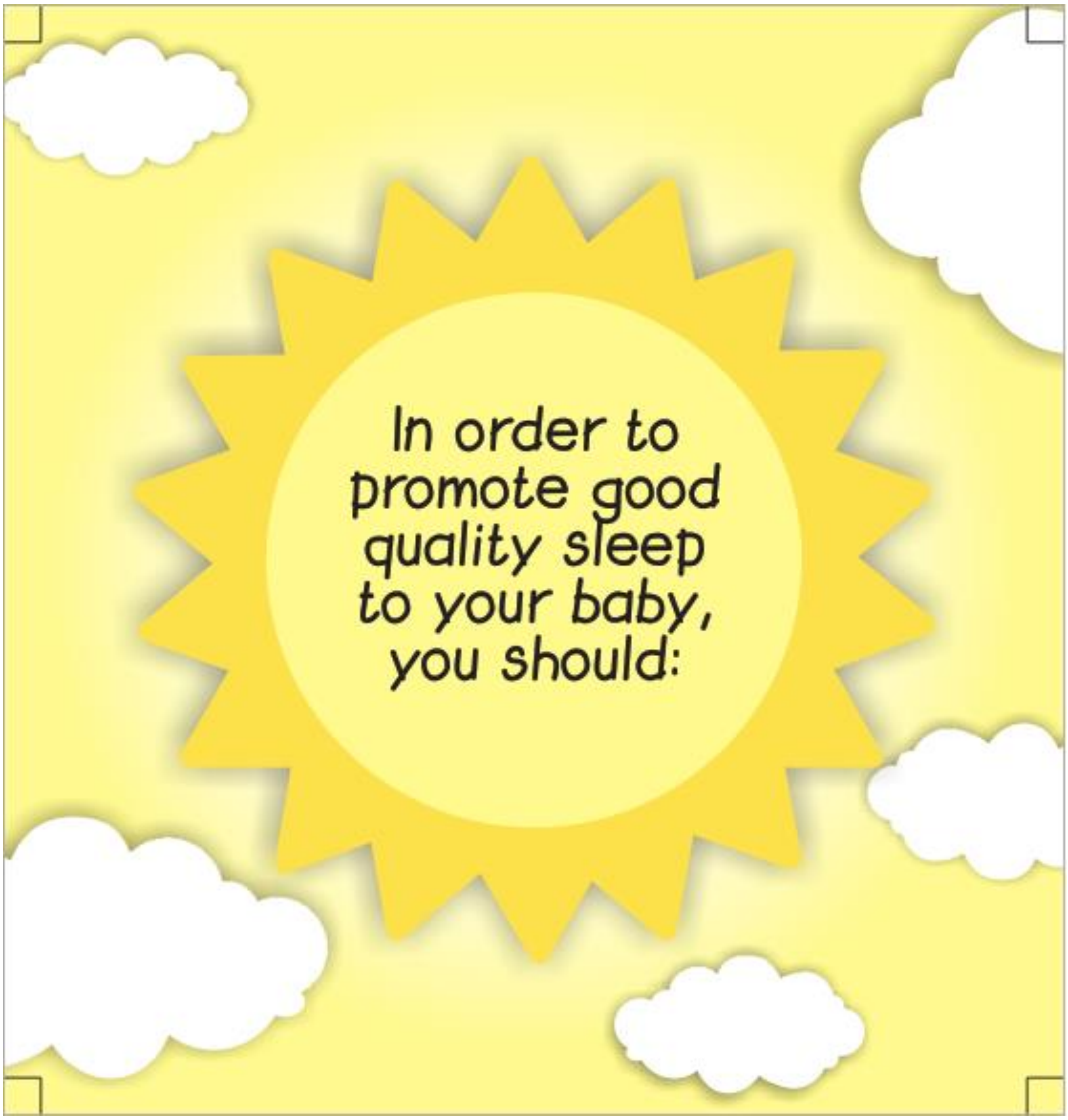

© 2019 Santos IS et al. *JAMA Network Open.*

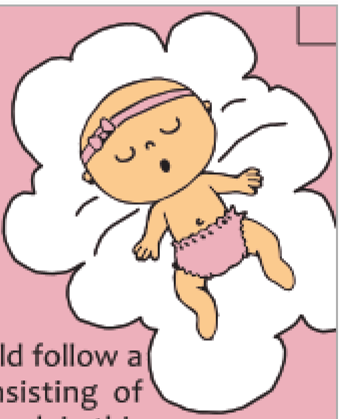

1 Have a routine leading up to sleep time

About **1 hour** before sleep time, you should follow a consistent routine with your child consisting of feeding, bathing, and putting him/her to bed, in this order. During this period, it is important for the home environment to be calm, with lights and noises kept down. By creating this consistent routine your baby will soon understand that the moment to sleep is approaching, making it easier for him/her to sleep faster and maintain a good-quality sleep.

In order to facilitate keeping this routine, always have this booklet at hand so you can consult it any time.

Remember to complete your child´s Sleep Diary; this will also help you stick to the routines.

© 2019 Santos IS et al. *JAMA Network Open.*

2 Prepare the environment to facilitate sleep

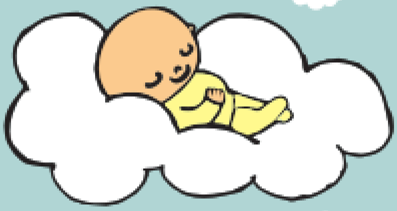

Your child´s bedroom should be kept with a dim light close to sleep time, and temperature must be mild. Activities that normally make your baby active must be avoided in the hour preceding sleep time, such as playing games that usually make your child overexcited.

3 Initiate sleep in the cot

Remember to place your baby drowsy but not asleep in his/her cot. This way your child will not associate falling asleep with being rocked or fed, therefore making it easier for him/her soothe him/herself back to sleep when waking during the night.

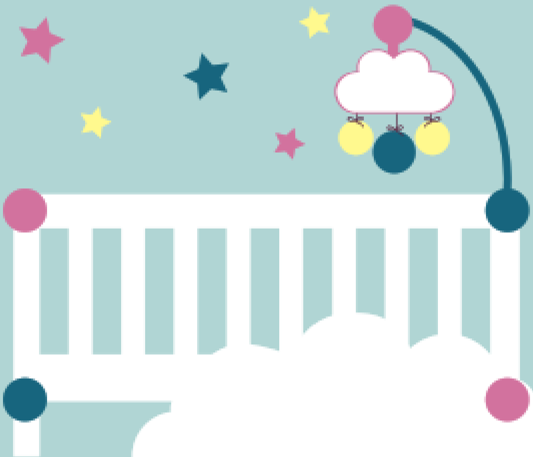

© 2019 Santos IS et al. *JAMA Network Open.*

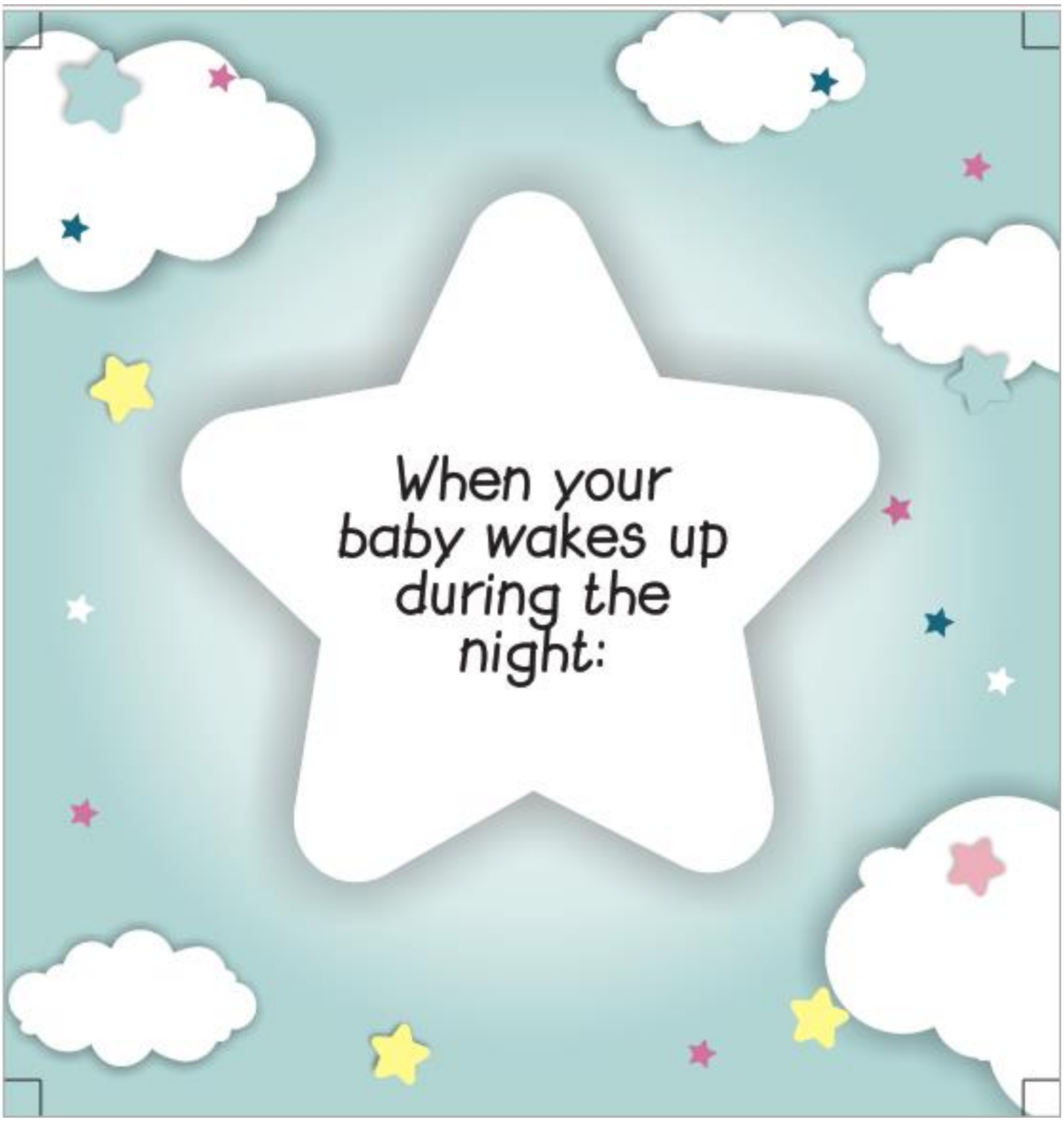

© 2019 Santos IS et al. *JAMA Network Open.*

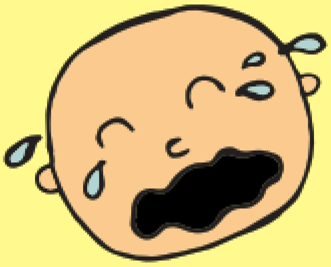

1) In the first night of the intervention, time 1 minute before responding to your baby´s crying. Add 30 seconds to the former evening timing in the following nights before responding to the crying.

2) Turn on low lights when attending to your baby. Avoid leaving the room too bright when responding to your baby´s night waking.

3) When attending to your baby, calm him/her down, cuddle him/her and reinforce that it is sleep time, laying him/her down as many times as needed for him/her to fall back asleep.

4) Your interventions must be short. Don´t wait for the baby to be asleep to return to your bed.

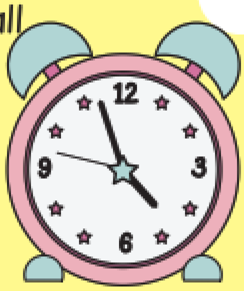

© 2019 Santos IS et al. *JAMA Network Open.*

Other important information

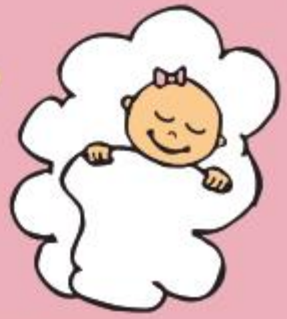

-The safest sleeping position for your baby is on his/her back.

-Breastfeeding is important to promote your child´s adequate development and growth, as well as establishing a unique bond between you and your baby e preventing against sudden infant death. You should not, however, breastfeed your baby to sleep. If he/she falls asleep while feeding, wake him/her up by changing the diaper or stimulating him/her gently, before putting him/her in the cot.

-Do not share the bed with your baby. Bed sharing increases the risk of suffocation.

-During the first six months of life, your baby can sleep on the same room as you, next to your bed, without sharing the bed with you.

-The mattress on which the baby sleeps should be firm. Cot bumpers or other objects kept inside the cot and on which the baby may suffocate must be avoided.

© 2019 Santos IS et al. *JAMA Network Open.*

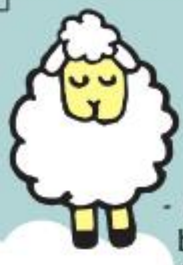

- Cot safety issues are important: distance between bars must be small, in order to keep your child from falling or placing his/her head between them. Lateral rolling must be protected so that your child is not able to lower it and fall. Keep the lateral bars high enough so that your child cannot jump over it. By guaranteeing a safe cot you will be able to put the oriented techniques into practice whilst keeping your baby safe from accidents.

- Do not cover your baby´s face with blankets or bed sheets. Don´t place objects or toys in the cot that could cover your baby´s face. Small toys should not be kept inside the cot, in order to avoid aspiration.

- Avoid over or under heating the baby; adapt his/her sleepwear to the environment´s temperature.

- Keep your house a smoke-free environment.

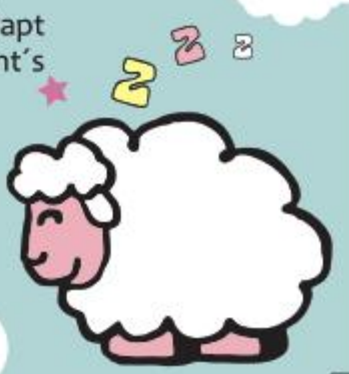

© 2019 Santos IS et al. *JAMA Network Open.*

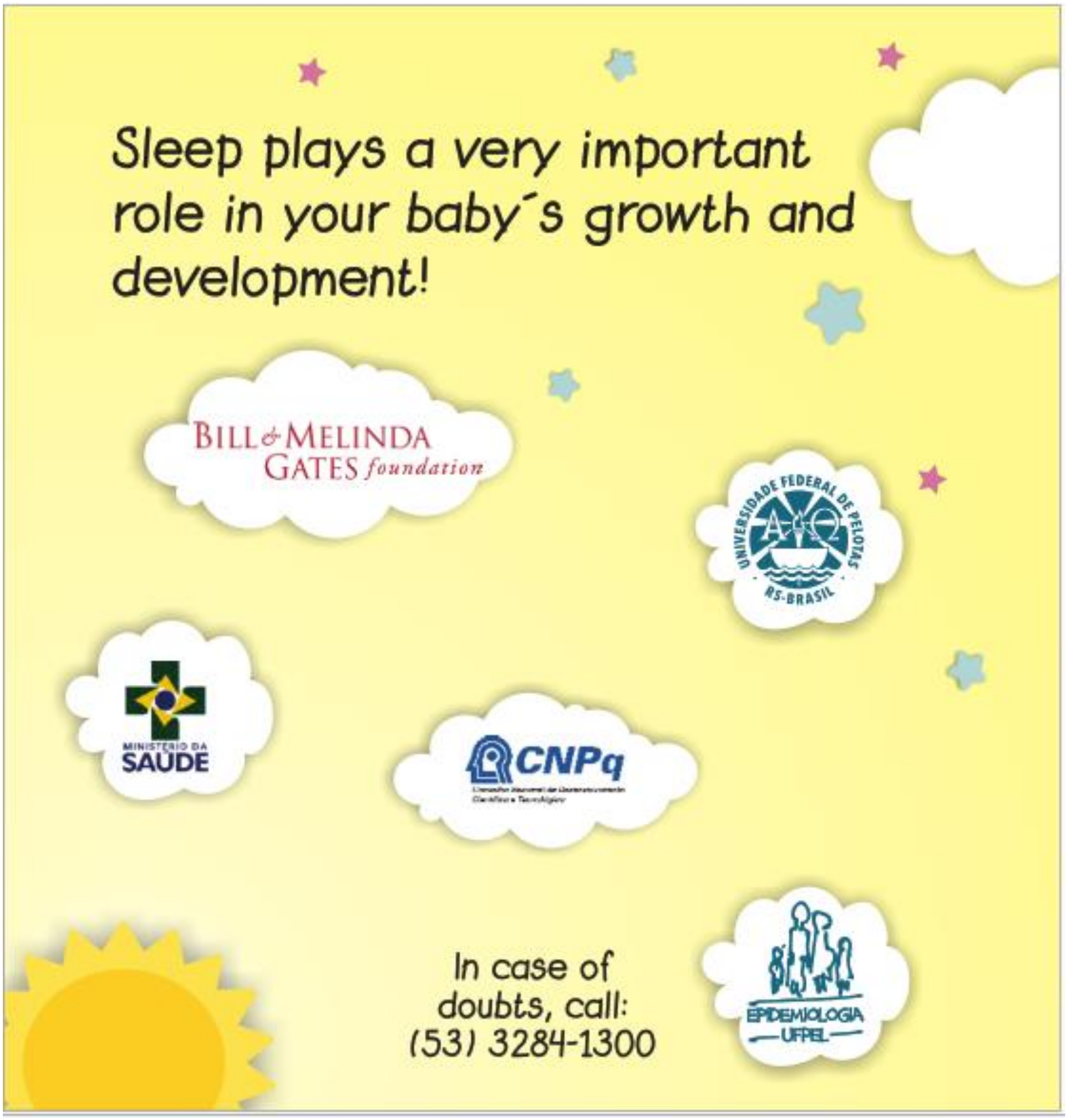

© 2019 Santos IS et al. *JAMA Network Open.*

2) Outliers definition according to child age:

- At six months of age: Total sleep duration ≥ 18 hours as obtained by interview, ≤ 5 hours as recorded in diaries or ≥ 18 hours as registered by actigraphy.

- At 12 months of age: Total sleep duration ≥ 16 hours by actigraphy.

- At 24 months: Total sleep duration ≤ 4 hours or ≥ 16 hours as obtained by interview

Number of children with unlikely total sleep duration:

At six months: N = 9 by interview, N = 4 by diaries and N= 4 by actigraphy

At 12 months: N = 2 by actigraphy

At months: N = 7 by interview and N = 2 by actigraphy

© 2019 Santos IS et al. *JAMA Network Open.*

eTable 1. Maternal Recall at 6 Months After Childbirth of the Advice Received at Baseline

Advice	N	%
Establishment of a night-time sleep routine (n=268)	157	58.6 %
Improvements in the environment to promote falling to sleep (n=269)	91	33.8%
Put the child to bed while still sleepy rather than when already asleep (n=268)	199	74.3%
Wait 1-2 minutes before attending to the child during nocturnal awakenings (n=265)	159	60.0%

© 2019 Santos IS et al. *JAMA Network Open.*

eTable 2. Effect of Intervention on Growth and Development at Age 12 mos

	**Intervention Mean (SD)**	**Control Mean (SD)**	**Difference (CI95%)**
Anthropometry	**N=290**	**N=281**	
Weight, mean (SD), kg	10.23 (1.39)	10.27 (1.54)	-4.52 (-0.28;0.20)
Length, mean (SD), cm	75.33 (3.43)	75.54 (3.24)	-0.21(-0.76;0.34)
Weight/Age z-score	0.71 (1.08)	0.71 (1.22)	-0.01 (-0.20;0.18)
Length/Age z-score	0.08 (1.28)	0.14 (1.28)	-0.06 (-0.27;0.15)
Body Mass Index/Age z-score	0.89 (1.24)	0.82 (1.29)	0.08 (-0.13;0.29)
Neurodevelopment (OX-NDA) [a]	**N=279**	**N=270**	
Overall score	70.78 (9.89)	69.39 (9.06)	1.39 (-0.20;2.99)
Cognitive domain	67.92 (11.16)	65.79 (10.45)	2.13 (0.31;3.95)
Motor domain	81.07 (13.16)	81.07 (12.18)	0.11 (-2.02;2.25)
Language domain	65.13 (14.46)	63.79 (14.91)	1.34 (-1.13;3.81)

[a]OX-NDA: Oxford Neurodevelopment Assessment tool

© 2019 Santos IS et al. *JAMA Network Open.*

eTable 3. Effect of Intervention on Growth and Development at Age 24 mos

	Intervention Mean (SD)	Control Mean (SD)	Difference (CI95%)
Anthropometry	**N=266**	**N=260**	
Weight, mean (SD), kg	12.61 (1.86)	12.50 (1.70)	0.11 (-0.20;0.42)
Length, mean (SD), cm	86.64 (3.85)	86.35 (3.70)	0.30 (-0.34;0.94)
Weight/Age z-score	0.53 (1.15)	0.44 (1.09)	0.09 (-0.11;0.28)
Length/Age z-score	0.01 (1.20)	-0.10 (1.17)	0.12 (-0.09;0.32)
Body Mass Index/Age z-score	0.72 (1.04)	0.73 (1.08)	-0.00 (-0.19;0.18)
Neurodevelopment (INTER-NDA) [a]	**N=267**	**N=262**	
Overall score	16.10 (1.63)	15.88 (1.92)	0.22 (-0.08;0.52)
Language domain	27.13 (7.16)	26.54 (7.14)	0.59 (-0.62;1.80)
Behavioural domain	8.54 (2.51)	8.83 (2.81)	-0.29 (-0.74;0.16)
Social domain	8.19 (2.94)	8.12 (3.04)	0.07 (-0.43;0.58)

[b]INTER-NDA: Intergowth 21st- Neurodevelopment Assessment tool

© 2019 Santos IS et al. *JAMA Network Open.*
