# Supplementary material for: Effect of Parental Counseling on Infants’ Healthy Sleep Habits in Brazil: A Randomized Clinical Trial
Source: JAMA Netw Open. 2019 Dec 20;2(12):e1918062. doi: 10.1001/jamanetworkopen.2019.18062 (PMC6991232; doi:10.1001/jamanetworkopen.2019.18062)
Supplement: Supplement 3. — Data Sharing Statement [file jamanetwopen-2-e1918062-s003.pdf]

Data Sharing Statement

Data

Data available: Yes
Data types: Deidentified participant data, Data dictionary
How to access data: Proposals should be directed to cpublicacoes.coortespelotas@gmail.com.
When available: With publication

Supporting Documents

Document types: None

Additional Information

Who can access the data: To get access, data requestors will need to sign a data access agreement.
Types of analyses: Data will be made available for any purpose.
Mechanisms of data availability: Data will be made available with investigator support after approval of a proposal and signature of data access agreement.
